# Spillover HIV prevention effects of a cash transfer trial in East Zimbabwe: evidence from a cluster-randomised trial and general-population survey

**DOI:** 10.1186/s12889-020-09667-5

**Published:** 2020-10-23

**Authors:** Robin Schaefer, Ranjeeta Thomas, Laura Robertson, Jeffrey W. Eaton, Phyllis Mushati, Constance Nyamukapa, Katharina Hauck, Simon Gregson

**Affiliations:** 1 MRC Centre for Global Infectious Disease Analysis, Department of Infectious Disease Epidemiology, Imperial College London, London, UK; 2 Department of Health Policy, London School of Economics and Political Science, London, UK; 3 Independent, London, UK; 4 Independent, Harare, Zimbabwe; 5 Biomedical Research and Training Institute, Harare, Zimbabwe

**Keywords:** Cash transfers, social protection, HIV prevention, sexual behaviour, school enrolment, mental distress, Zimbabwe

## Abstract

**Background:**

Benefits of cash transfers (CTs) for HIV prevention have been demonstrated largely in purposively designed trials, commonly focusing on young women. It is less clear if CT interventions not designed for HIV prevention can have HIV-specific effects, including adverse effects. The cluster-randomised Manicaland Cash Transfer Trial (2010–11) evaluated effects of CTs on children’s (2–17 years) development in eastern Zimbabwe. We evaluated whether this CT intervention with no HIV-specific objectives had unintended HIV prevention spillover effects (externalities).

**Methods:**

Data on 2909 individuals (15–54 years) living in trial households were taken from a general-population survey, conducted simultaneously in the same communities as the Manicaland Trial. Average treatment effects (ATEs) of CTs on sexual behaviour (any recent sex, condom use, multiple partners) and secondary outcomes (mental distress, school enrolment, and alcohol/cigarette/drug consumption) were estimated using mixed-effects logistic regressions (random effects for study site and intervention cluster), by sex and age group (15–29; 30–54 years). Outcomes were also evaluated with a larger synthetic comparison group created through propensity score matching.

**Results:**

CTs did not affect sexual debut but reduced having any recent sex (past 30 days) among young males (ATE: − 11.7 percentage points [PP] [95% confidence interval: -26.0PP, 2.61PP]) and females (− 5.68PP [− 15.7PP, 4.34PP]), with similar but less uncertain estimates when compared against the synthetic comparison group (males: -9.68PP [− 13.1PP, − 6.30PP]; females: -8.77PP [− 16.3PP, − 1.23PP]). There were no effects among older individuals. Young (but not older) males receiving CTs reported increased multiple partnerships (8.49PP [− 5.40PP, 22.4PP]; synthetic comparison: 10.3PP (1.27PP, 19.2PP). No impact on alcohol, cigarette, or drug consumption was found. There are indications that CTs reduced psychological distress among young people, although impacts were small. CTs increased school enrolment in males (11.5PP [3.05PP, 19.9PP]). Analyses with the synthetic comparison group (but not the original control group) further indicated increased school enrolment among females (5.50PP [1.62PP, 9.37PP]) and condom use among younger and older women receiving CTs (9.38PP [5.90PP, 12.9PP]; 5.95PP [1.46PP, 10.4PP]).

**Conclusions:**

Non-HIV-prevention CT interventions can have HIV prevention outcomes, including reduced sexual activity among young people and increased multiple partnerships among young men. No effects on sexual debut or alcohol, cigarette, or drug consumption were observed. A broad approach is necessary to evaluate CT interventions to capture unintended outcomes, particularly in economic evaluations.

**Trial registration:**

ClinicalTrials.gov, NCT00966849. Registered August 27, 2009.

## Introduction

Poverty and inequality in wealth and resources are important social determinants of health [1]. Cash transfers (CTs) have a long history as a social protection policy and government-led CT programmes have been implemented to reduce poverty and vulnerability in many low- and middle-income countries, including more recently in sub-Saharan Africa [2, 3]. CTs are the direct transfer of money to an eligible person or household. Most CT programmes involve regular rather than one-off payments of varying amounts, often adjusted for other household factors such as number of children. CTs may be unconditional (UCTs) or conditional (CCTs) on meeting specific criteria. They have been demonstrated to have a range of positive impacts on health, nutritional, and educational outcomes [4]. Recently, interests increased in CTs as a strategy for prevention of HIV and other sexually transmitted infections (STIs) [5, 6].

CTs can have a range of effects on behaviour relevant for HIV/STI prevention. If CTs are conditional on sexual decisions, including staying free of STIs, CCTs create a price effect by making sexual behaviour associated with STI risks costlier as income is lost when not meeting these conditions [7]. CCTs also bring the positive effects of safer sexual behaviour closer in time, addressing the discounting of future benefits (staying free of HIV/STIs) compared to present benefits of potentially unsafe behaviour [7]. Moreover, CTs have an income effect and can address social determinants of HIV/STI infection risks by improving economic positions of individuals and so abilities to engage in safer sexual behaviour. This income effect may be particularly relevant for women who are often unable to negotiate condom use in sexual relationships that are characterised by economic imbalances and may have economic motives to engage in transactional relationships or sex work. Improved incomes may also remove economic barriers to accessing healthcare and HIV/STI prevention methods [8].

Benefits of CTs for HIV/STI prevention have been examined in randomised controlled trials (RCTs) [8, 9] and evaluations of national CT programmes [10–14], as summarised in Tables 1 and 2 and with more details provided in Additional file 1. Among school children in South Africa, one RCT (CAPRISA 007) found effects of CTs on HIV herpes simplex virus type 2 (HSV-2) incidence [15], while the HPTN 068 study did not find such effects [16], although HPTN 068 found reduced numbers of sexual partners and delayed sexual debut [17]. Other RCTs also found effects on sexual behaviour [18, 19], although some of these effects were found to be short-lived [20] . Similarly, RCTs with CTs conditional on staying STI-free showed effects on STI prevalence but these effects tended to disappear after the end of the studies [21–23]. In addition to purposively designed trials, evaluations of CT programmes in South Africa [10, 11], Kenya [13, 14, 24], and Malawi [12] found effects on sexual behaviour among younger people relevant for HIV/STI prevention, including reductions in transactional and age-disparate relationships and delayed sexual debut.

**Table 1:** Findings of previous evaluations of the effects of cash transfers on HIV prevention outcomes in sub-Saharan Africa among females

Evaluation	Ref	Evaluated population ^**a**^	CT type	Biological outcomes	Sexual debut, frequency of sex, number of partners	Condom use, condomless sex	Other
South Africa							
HPTN 068	[16] [17]	School: 13–20 years	CCT	No effect on HIV or HSV-2 incidence	Reduced sexual debut; fewer partners; no effect multiple partners	Reduced unprotected sex	Reduced IPV; no effect on age-disparate relationships or transactional sex
CAPRISA 007	[15]	School: grade 9/10	CCT	Reduced HSV-2 incidence	Not measured	Not measured	Not measured
NSP (national)	[10]	10–18 years	UCT	Not measured	No effect multiple partners	No effect unprotected sex	Reduced age-disparate relationships and transactional sex
CSG (national)	[11]	15–16 years	UCT	Not measured	Reduced sexual debut; fewer partners	Not measured	Not measured
Malawi							
SIHR	[18]	School: 13–22 years	UCT, CCT	Reduced HIV & HSV-2 prevalence	No effect sexual debut, reduced frequency of sex past week	No effect unprotected sex	Reduced age-disparate relationships
SCT (national) 24-month	[12]	13–19 years	UCT	Not measured	No effect sexual debut; reduced sex acts ^b^	No effect condom use ^b^	Reduced age-disparate relationships; no effect on transactional sex; reduced IPV
MIP	[20]	15+ years	CCT	Not measured	Reduced sex past 9 days	No effect condom use	Net decrease condomless sex
Zimbabwe							
HSCT (national) 48-month ^c^	[25]	13–20 years	UCT	Not measured	Increased age at first sex; no effect number of sex acts or partners	Reduced unprotected sex	No effect on transactional sex
Kenya							
CT-OVC (national)	[13]	15–25 years	UCT	Not measured	Reduced sexual debut; reduced multiple partners	No effect condom use	No effect on transactional sex
Tanzania							
RESPECT	[21]	18–30 years	CCT	Reduced STI prevalence	Reduced multiple partners	No effect condom use	Not measured
Lesotho							
Lottery	[22]	18–32 years	CCT	Reduced HIV incidence	Reduced number of partners ^b^	Reduced unprotected sex ^b^	Not measured

*Ref* Reference, *CT* Cash transfer, *UCT* Unconditional cash transfer, *CCT* Conditional cash transfers, *HSV-2* Herpes simplex virus type 2, *IPV* Intimate partner violence

Study and programme names in South Africa are the HIV prevention trial network (HPTN) 068 study, and the national social protection (NSP) and child support grant (CSG) programmes. In Malawi they are the Schooling, Income, and Health Risk (SIHR) study, the Social Cash Transfer (SCT) programme, and the Malawi Incentives Programme (MIP). In Zimbabwe it is the Harmonised Social Cash Transfer (HSCT) programme. In Kenya it is the Cash Transfer for Orphans and Vulnerable Children (CT-OVC) programme

^a^ If the evaluation included a follow-up, the age of the population refers to baseline eligibility criteria

^b^ Both sexes evaluated together

^c^ The evaluation of the HSCT programme in Zimbabwe after 12 months had a small sample size, so only results from the 48-month evaluation are reported

**Table 2:** Findings of previous evaluations of the effects of cash transfers on HIV prevention outcomes in sub-Saharan Africa among males

Evaluation	Ref	Evaluated population ^**a**^	CT type	Biological outcomes	Sexual debut, frequency of sex, number of partners	Condom use, condomless sex	Other
South Africa							
CAPRISA 007	[15]	School: grade 9/10	CCT	Reduced HSV-2 incidence	Not measured	Not measured	Not measured
NSP (national)	[10]	10–18 years	UCT	Not measured	Reduced multiple partners	No effect unprotected sex	No effect on age-disparate relationships or transactional sex
CSG (national)	[11]	15–16 years	UCT	Not measured	No effect sexual debut; fewer partners	Not measured	Not measured
Malawi							
SCT (national) 24-month	[12]	13–19 years	UCT	Not measured	Reduced sexual debut; reduced sex acts ^b^	No effect condom use ^b^	Not measured
MIP	[20]	15+ years	CCT	Not measured	Increased sex past 9 days	Increased condom use	Net increase condomless sex
Zimbabwe							
HSCT (national) 48-month ^c^	[25]	13–20 years	UCT	Not measured	Reduced sexual debut; no effect number of sex acts or partners	Reduced unprotected sex	No effect on transactional sex
Kenya							
CT-OVC (national)	[13]	15–25 years	UCT	Not measured	Reduced sexual debut; no effect multiple partners	No effect condom use	No effect on transactional sex
Tanzania							
RESPECT	[21]	18–30 years	CCT	Reduced STI prevalence	No effect multiple partners	Increase condom use	Not measured
Lesotho							
Lottery	[22]	18–32 years	CCT	Reduced HIV incidence	Reduced number of partners ^b^	Reduced unprotected sex ^b^	Not measured

*Ref *Reference,* CT* Cash transfer, *UCT* Unconditional cash transfer, *CCT* Conditional cash transfers, *HSV-2* Herpes simplex virus type 2, *IPV* Intimate partner violence

Study and programme names in South Africa are the national social protection (NSP) and child support grant (CSG) programmes. In Malawi they are the Social Cash Transfer (SCT) programme and the Malawi Incentives Programme (MIP). In Zimbabwe it is the Harmonised Social Cash Transfer (HSCT) programme. In Kenya it is the Cash Transfer for Orphans and Vulnerable Children (CT-OVC) programme

^a^ If the evaluation included a follow-up, the age of the population refers to baseline eligibility criteria

^b^ Both sexes evaluated together

^c^ The evaluation of the HSCT programme in Zimbabwe after 12 months had a small sample size, so only results from the 48-month evaluation are reported

These evaluations of CTs in trials or national programmes demonstrate that CTs can have effects on sexual behaviour that are associated with reduced risks for HIV/STI infection, although few studies demonstrate effects on biological outcomes. There are further questions regarding the sustainability of the effects of CTs on behaviour and causal pathways of these effects [26]. Trials on CTs tended to focus on increasing school enrolment [15, 16, 18], while one study of the impact of a Kenyan UCT programme indicated that observed effects were only partially mediated by increased school enrolment [27]. A major limitation of studies of CTs is the focus on younger people, particularly young females, with effects among males being less clear. This is particularly relevant in the context of potential adverse effects of CTs in terms of possibly health-damaging behaviours, including risky sex and alcohol, cigarette, and drug consumption, which may be more common among males. While the HPTN 068 trial and two evaluations of South African CT programmes found no such adverse effects [10, 11, 28], evaluations of CTs in Kenya and Malawi found indications for increased unprotected sex among males receiving CTs [14, 20], and, in a qualitative study of a pilot CT programme in Johannesburg, participants reported that spending on drugs and alcohol was common among CT recipients and that some males engaged in criminal activities after the trial ended to compensate for the reduced income [29].

Most evidence for the effectiveness of using CTs for HIV/STI prevention comes from purposively designed trials [15, 20]. There is limited evidence about whether CT not designed for HIV prevention can have spillover effects (externalities of the intervention) that may be beneficial (e.g. partner reduction or increased condom use) or detrimental for HIV/STI prevention (e.g. increased condomless sex or alcohol use). Such spillover effects may occur when CTs have unintended effects on the individual receiving the CT or among individuals living in a household receiving CTs. Evaluations of national CT programmes consider such unintended spillover effects, but these have only been conducted in a limited number of settings. CT interventions, particularly CCT interventions, are associated with high costs, and the cost-effectiveness of these interventions could be increased – if there are beneficial spillover effects – or decreased. This cost-effectiveness is relevant for questions about the scalability of CT programmes [30]. Given the continuing interest of policy makers in CT as a social protection policy and for HIV/STI prevention, further evaluations of spillover effects of CTs in different settings are needed urgently.

### Poverty and CTs in Zimbabwe

Zimbabwe has experienced considerable economic decline since the late 1990s and increasing proportions of individuals living in poverty [31, 32]. This economic collapse followed shortly after the peak of one of the largest HIV epidemics in the world, which had considerable impacts on mortality [33] and led to a rapid increase in the number of children under the age of 18 years orphaned due to AIDS-related deaths of their parents, peaking at 880,000 in the period of 2005 to 2007 [34]. Rising poverty and food insecurity, particularly in rural areas, and increasing numbers of orphans and vulnerable children (OVC) led to growing interest in CTs as a social protection policy in Zimbabwe. Before rolling out a national CT programme to improve children’s development, a CT intervention was piloted in the Manicaland Cash Transfer Trial (Manicaland Trial), a cluster-randomised controlled trial (cRCT) in Manicaland, eastern Zimbabwe, which evaluated effects of CCTs and UCTs given to households on children’s (2–17 years) birth registration, immunisation, and school attendance [35].

The Manicaland Trial was terminated after 1 year due to plans to roll-out a national CT programme targeting vulnerable households. This Harmonised Social Cash Transfer (HSCT) programme was started to be implemented by the Ministry of Public Service, Labour and Social Welfare (MPSLSW) in 2011in 10 districts across Zimbabwe [36]. The HSCT programme involves unconditional monthly transfers of US$10–25. By 2014, 55,509 households were enrolled in 20 districts Zimbabwe [36]. Despite the long-term goal to cover over 250,000 households in all 65 districts of Zimbabwe, the number of enrolled households nearly halved by 2017 compared to 2014 [25].

The HSCT programme was evaluated using a non-experimental study design that involved a baseline survey in 2013 and follow-up surveys after 12 and 48 months [25, 36], reflecting different levels of exposure to the programme. One district in which implementation of the HSCT programme had started was selected in each of the three provinces. Control households were selected from three districts neighbouring the three treatment districts where the HSCT programme was planned to be rolled out at a later stage. These districts were matched on agro-ecological, cultural, and developmental characteristics by MPSLSW experts. The short- and long-term evaluations of the HSCT found no improvements in primary or secondary school enrolment or grade progression [25, 36]. In the long-term evaluation, among youth aged 13–20 years, a 9% reduction in sexual debut and a 5% reduction in unprotected sex in the past 3 months was found in households receiving CTs, although no effects of CTs on number of sexual acts or sexual partners were found [25].

### Objectives of this study

While the HSCT programme evaluation in Zimbabwe indicated that CTs can have impacts on young people that are likely to be beneficial for HIV prevention, the non-experimental evaluation approach has limitations and trends in outcome measures in control and treatment clusters may not be comparable (for example, the evaluations found significant differences in school attendance, expenditure on healthcare, and perceptions about HIV infection risks in the treatment and control households at baseline [36]). Therefore, such an approach is inferior to experimental or quasi-experimental evaluation methods. Moreover, the HSCT evaluation suffers from limitations similar to other CT evaluations, focusing on sexual behaviour among young people and excluding older individuals.

Given that the Manicaland Cash Transfer Trial was conducted in the same communities as the Manicaland General-Population Cohort (Manicaland Cohort), a long-term open-cohort study, additional information was collected on members of households receiving CTs through ongoing longitudinal surveys of the population. This provided a unique opportunity for evaluating spillover effects of a CT intervention not designed for HIV prevention. This study expands the literature on CTs for HIV prevention by focusing on:

1. Spillover effects on outcomes relevant for HIV/STI prevention, including direct effects on sexual behaviour and on possible mediating factors such as mental health, schooling, and use of alcohol and drugs; and
2. Whether effects differed between sub-groups, including younger and older people, males and females, and individuals living in male- and female-headed households.

## Methods

### Setting and data

#### Manicaland province in Zimbabwe

This study was based on data collected in Manicaland, eastern Zimbabwe. This province is characterised by largely rural communities and has poorer educational and population health outcomes than other provinces [37, 38]. Adult (15+ years) HIV prevalence declined from peaks of over 25% in the late 1990s to 11% in 2015–16 [39], the lowest level of any province in Zimbabwe, but the number of people living with HIV in Manicaland is one of the highest in the country [40]. Manicaland has been identified as a ‘hotspot’ of HIV transmission [40], making it a priority in the Zimbabwe National HIV and AIDS Strategic Plan [41], and as a priority for the introduction of pre-exposure prophylaxis (PrEP) for key populations by the DREAMS programme in Zimbabwe [42].

#### The Manicaland Cash Transfer Trial (Manicaland Trial)

The Manicaland Trial was a cRCT testing the effects of CCTs and UCTs on children’s (2–17 years) development [35]. Ten sites, representing small towns, subsistence farming areas, agricultural estates, and roadside business centres, were chosen for the CT intervention among existing Manicaland Cohort study areas. A baseline household survey was conducted between July and September 2009 and information on all household members was obtained from the most senior member of the household present at the time of the survey. The survey identified 11,820 households with 63,065 household members (all ages). Of these, 4043 households with 22,525 household members were found eligible for the Trial. Eligible households included those that had a head of household under 18 years of age, cared for an orphan or disabled or chronically ill person, or were in the poorest 20% of a household asset-based wealth index. Community groups were also involved to verify selections of the poorest households.

Each of the 10 sites was divided into three homogenous clusters, which were randomly assigned to UCT, CCT, or control. Eligible households in treatment clusters were enrolled in the CT programmes, which included six cash disbursements from January 2010 to January 2011. In the UCT programme, households collected $18 plus $4 for each child in the household (up to a maximum of $30) from pay points every 2 months. In the CCT programme, households received the same amount if they met several conditions: applied for a birth certificate for children not yet registered within 3 months; kept children under 5 years up-to-date with vaccinations and attended growth-monitoring sessions twice a year; children aged 6–17 years attended school at least 90% of the time in a month; and one person from the household attended at least two-thirds of a local parenting skills class. The conditions in the CCT programme were not enforced for the first 6 months and, after that period, when not meeting the conditions, support was provided and CTs were reduced but not withheld, so the CCT intervention was ‘soft’ and similar to the UCT. All households, including in control sites, received standard agricultural packages with seeds and fertilisers. Parenting skill classes were provided in all clusters.

The Trial was terminated after 1 year due to funding constraints and plans of the Zimbabwean government to roll out a national CT programme. A follow-up survey was conducted between March and May 2011. Of 4043 households enrolled into the Trial at baseline, 3818 (94.4%) were followed up, with the majority of loss-to-follow-up due to out-migration. Ethical approval for the Manicaland Trial was obtained by the Imperial College London Research Ethics Committee and the Medical Research Council of Zimbabwe. The Manicaland Trial is registered with ClinicalTrials.gov (NCT00966849) and more details are provided elsewhere [35].

#### The Manicaland General-Population Cohort (Manicaland Cohort)

**Fig. 1:**
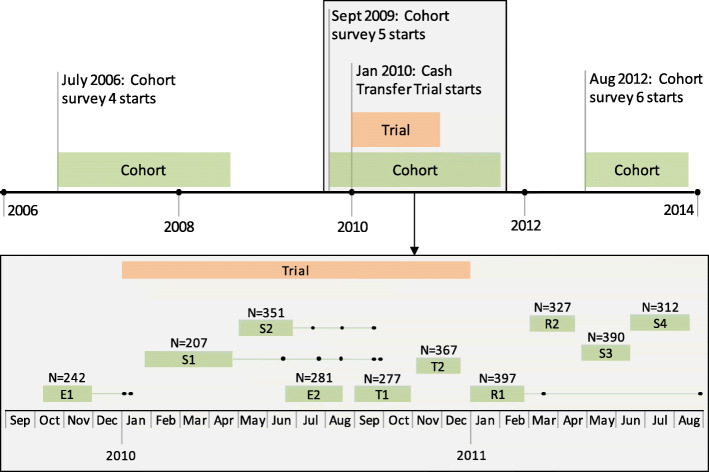
Timeline of data collection for the Manicaland Trial and Cohort, including for different study sites. Study site types are: Farming estates (E), subsistence farming areas (S), small towns (T), and roadside business centres (R). Numbers indicate different study sites of the same type. Dots indicate where individuals from a study site participated at a later date than the majority of individuals from the study sites

The Manicaland Trial was conducted in the 10 of 12 study sites in which the Manicaland Cohort completed six surveys of a general-population open-cohort study between 1998 and 2013. This study used data from survey five of the Cohort, conducted between September 2009 and August 2011, with one study site covered in the Manicaland Cohort before the start of the Trial (Fig. 1). A household census enlisted 13,180 households (98% response rate) from which 11,187 individuals aged 15–54 years were included in the survey (78.9% response rate). A face-to-face interview was conducted with each study participant individually by an interviewer of the same sex and in the local language (Shona), collecting information on socio-demographic factors, behaviour, and HIV-specific perceptions. HIV status was determined on a dried blood spot sample. Ethical approval for the Manicaland Cohort was provided by the Imperial College London Research Ethics Committee and the Medical Research Council of Zimbabwe. More details are provided elsewhere [43].

#### Data preparation

**Fig. 2:**
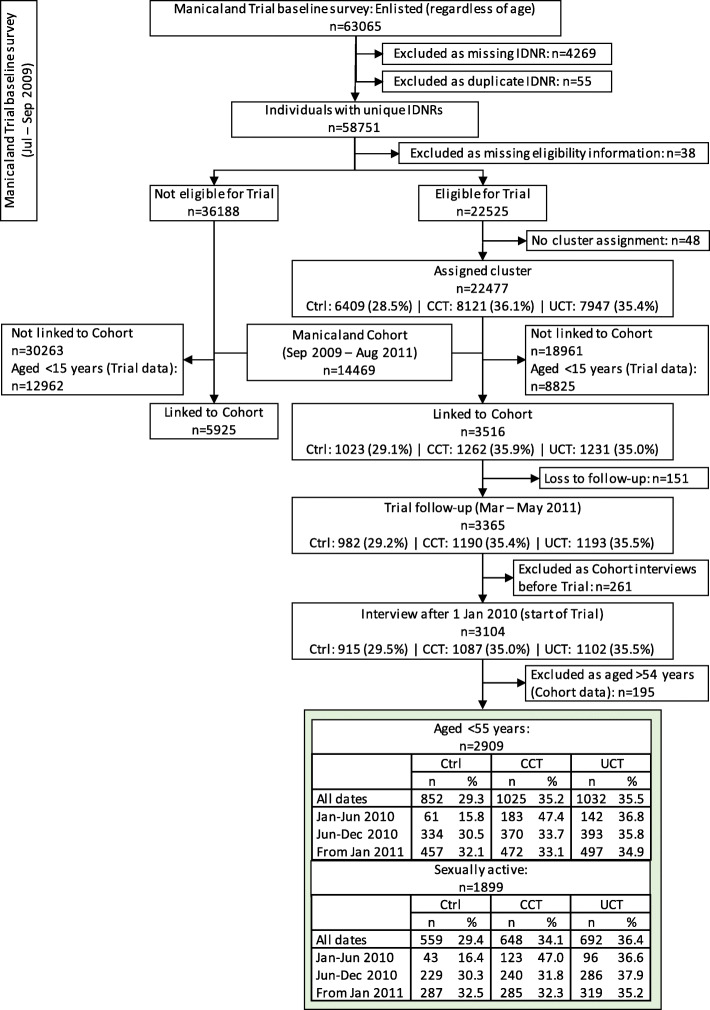
Preparation of data combining the Manicaland Trial and Cohort. Numbers of participants (n) refer to individuals. In the Manicaland Cash Transfer Trial surveys, the number of individuals were those reported to live in a household by a senior member of the household. The sample for this analysis is displayed in the green box. The dates in the green box indicate the number of individuals included in the Manicaland Cohort at different time periods of the Manicaland Trial (early during the Trial: January to June 2010; later during the Trial: June to December 2010; after the Trial ended: from 2011). Abbreviations refer to the different Manicaland Trial groups: Control (Ctrl), conditional cash transfer (CCT), and unconditional cash transfer (UCT). IDNR refers to unique study identifier number

This analysis was based on Manicaland Cohort data available for individuals living in households that participated in the Manicaland Trial. The linking the Manicaland Trial and Cohort data is described in Fig. 2. Of those individuals enlisted in the baseline survey of the Trial and identified as eligible, 3516 individuals were linked to the Cohort data. Individuals living in study site one (a tea estate) (*n* = 261) were excluded as this study site was covered by the Cohort survey before the Trial started (Fig. 1). Moreover, the analysis was restricted to those aged under 55 years, leaving 2909 individuals as the analysed sample (of which 1899 reported that they had their sexual debut). Of these, 1570 individuals were aged 15–29 years (577 CCT, 536 UCT, and 457 control) and 1339 aged 30–54 years (448 CCT, 496 UCT, 395 control) (see Additional file 2, section 3, for details on the socio-economic characteristics of the population). The study design of using data on outcomes from the Manicaland Cohort is likely to reduce impacts of reporting bias as it was collected independently of the Manicaland Trial.

Data from the Trial baseline survey were used to evaluate whether there were systematic differences in socio-economic characteristics between individuals aged 15–54 years eligible for the Trial linked to the Cohort and those not linked. This comparison indicated that individuals linked to the Cohort (included in this analysis) were older, more likely to be female and enrolled in school, and of lower socio-economic status than those not linked to the Cohort. However, these differences were similar for the control and treatment groups of the Trial, suggesting that no selection bias was introduced. See Additional file 2 (section 2) for details. All other analysed data in this study were taken from the Manicaland Cohort survey (not the Trial surveys).

### Theoretical framework and measures

#### Hypothesised effects and outcome and treatment measures

**Fig. 3:**
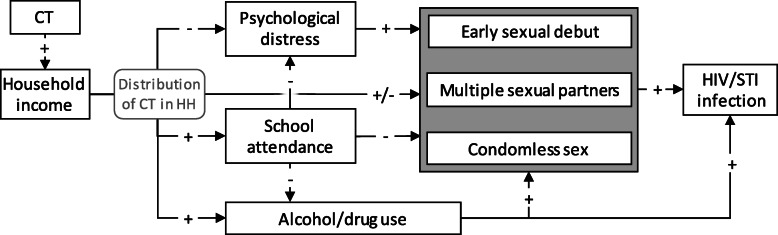
Theoretical framework of expected effects of cash transfers (CTs). Expected effects of CTs on sexual behaviour (grey box) and other outcomes linked to sexual behaviour and risks for infection with HIV and sexually transmitted infections (STIs), including substance use, school attendance, and psychological distress. Signs indicate expectations of increasing (+) or decreasing (−) effects. The hypothesised effect of school attendance is not applicable to older individuals (above the age of 20 years in this analysis). The distribution of CTs within households (HH) was unknown and some effects are expected to differ by sex (+/−) and type of household (male- or female-headed). See Table 3 for further information

Expected effects of CTs on outcomes in this analysis are illustrated in Fig. 3 and described in Table 3. CTs were hypothesised to impact sexual behaviour directly through improved income and economic empowerment. Given the short duration of the Manicaland Trial, no measurable effects on biological outcomes (HIV infection or pregnancy) were expected, so primary outcomes of this analysis related to sexual behaviour (Table 3). Secondary outcomes, analysed for their potential in mediating effects on sexual behaviour (Fig. 3), included school enrolment [44, 45], psychological distress [46], and consumption of alcohol, cigarettes, and drugs. See Additional file 2 (section 1) for information on data for measures.

**Table 3:** Primary and secondary outcomes in this analysis and their measurements, populations for which they were considered, and hypothesised links to cash transfers

Outcome	Measurement	Population	Hypothesised links to CTs
**Primary outcomes**			
Any recent sexual activity	Had sexual intercourse in the past 30 days	M & F (15–29) M & F (30–54) (who had sex before)	Females: CTs may lead to economic empowerment and reduce the economic necessity to engage in transactional relationships or sex work, which may be reflected in lower numbers of sexual partners and having recent sexual intercourse. Increased school enrolment (and so attendance) may reduce sexual activity and numbers of partners. Similarly, CTs may reduce economic barriers to accessing condoms and economic dependence on sexual partners, so condom use may be more viable. Males: Increased sexual activity and number of partners and reduced condom use may be adverse effects of the economic empowerment through CTs, while increased school enrolment (and attendance) may reduce sexual activity.
Multiple sexual partners	> 1 sexual partner in past 12 months		
Condom use	Condom use during last sexual intercourse		
Sexual debut	Having had sex before	M & F (15–20)^a^	CTs may lead to delayed sexual debut partially through improved school enrolment and by reducing economic incentives to engage in sexual activity (for females).
**Secondary outcomes**			
Psychological distress	≥7 symptoms of psychological distress (25-item scale)	M & F (15–29) M & F (30–54)	Poverty and income inequality can lead to psychological distress, which may be alleviated with CTs. See [46] for details.
School enrolment	Currently enrolled in schoolb	M & F (15–20)^c^	CTs remove economic barriers to school enrolment. School attendance was also a condition in the conditional CT intervention.
Alcohol use	Visited a beer hall in past 30 days or having > 3 drinks when drinking alcohol	M & F (15–29) M & F (30–54)	Adverse effects of CTs could include non-sexual potentially health-damaging behaviours, including alcohol and drug use and cigarette smoking. Alcohol and drug use could also lead to increase in potentially risky sexual behaviour.
Smoking	Current smoking of cigarettes		
Drug use	Taking any type of recreational drug		

*CTs* Cash transfers, *M* Males, *F* Females

Numbers after populations refer to age range in years. Data on all measures were taken from the Manicaland Cohort study. See Additional file 2 (section 1) for details on data and measurements.

^a^ Sexual debut was evaluated among those aged 15–20 instead of all young people because most young people will have had their sexual debut by the age of 20

^b^ The original Manicaland Trial measured school attendance, not enrolment, which is not directly comparable to the measure of school enrolment in the Manicaland Cohort

^c^ School enrolment was evaluated among those aged 15–20 instead of all young people because it is not applicable to older people

CTs in the Manicaland Trial were provided to the household, not focused specifically on individuals identified as at risk for HIV infection, and were not conditional on meeting HIV/STI-specific criteria. The conditions of the CCT intervention were ‘soft’, so effects on sexual behaviour were hypothesised to be similar in the CCT and UCT groups, except for school enrolment as school attendance was a condition of the CCT Trial group. As school enrolment may mediate effects between CTs and sexual behaviour, there may be differential effects in the CCT and UCT groups among younger people. Therefore, main analyses grouped CCTs and UCTs together, while sub-analyses tested for differences by CT type (see below).

### Analysis

Individual-level data from the Manicaland Trial were analysed using mixed-effects regressions [47], estimating effects of CTs on outcomes with fixed effects for Trial group assignment (treatment vs. control) and random effects for study site and Trial cluster (random intercepts). Gauss-Hermite quadrature was used to estimate parameters [48]. Average treatment effects (ATEs) were based on adjusted coefficients estimated by mixed-effects regression models for binary outcomes (instead of crude differences in means between treatment and control). ATEs represent absolute differences in percentages between Trial groups and are referred to as percentage point (PP) change; percentage (%) is used to refer to proportions.

Mixed-effects models with random effects for study site and cluster account for the clustering of individual survey responses within clusters and study sites [47]. Performing cluster-level analyses of aggregated data is most robust but not statistically efficient [49], particularly when clusters vary considerably in size, as for the Manicaland Trial. It was considered appropriate to analyse individual-level data as there were 27 clusters in this analysis of the Manicaland Trial, more than the minimum recommended by Hayes and Moulton [49].

#### Analyses of the original trial sample

ATEs of CTs were based on mixed-effects logistic regressions estimating associations between receiving CT (vs. control) and outcomes listed in Table 3. Separate models were estimated by sex and age group (young people [15–29 years]; older people [30–54]. Analyses of primary outcomes were restricted to those sexually active; analyses of secondary outcomes included all individuals.

Given that data on outcomes were taken from the Manicaland Cohort and were only available after the Trial started, balance in outcomes before Trial commencement could not be evaluated. Instead, to evaluate cluster balance, Trial groups were described in terms of socio-economic characteristics that were unlikely to be affected by the intervention or change within short time periods (age, sex, education, marital status, and socio-economic status) and HIV prevalence (which was not expected to be affected within the short period of the Trial) (see Additional file 2, section 3).

#### Analyses with synthetic comparison groups

To overcome limitations in sample size, a quasi-experimental approach utilised the large Manicaland Cohort data set, in addition to analyses of the original Trial sample. Propensity score matching (PSM) is a widely used method to create synthetic control groups matched to individuals who received an intervention in observational studies without randomised control groups. This allows for estimating treatment effects on outcomes and reduces effects of confounding [50, 51] (see Additional file 2, section 4, for more information on this method). PSM was used to create synthetic comparison groups from Cohort participants that did not live in Trial households but who participated in the coinciding survey of the Manicaland Cohort study. Propensity scores (PS) were estimated using logistic regressions including a set of background characteristics that were found to optimally balance the treatment and synthetic comparison group in preliminary analyses (age, marital status, socio-economic status, and HIV status). PSM was implemented separately for each study site (see [52–54]) and by sex and the same age groups (15–29; 30–54 years). After establishing the synthetic comparison group, the same analyses were performed as for the original Trial data (for a discussion of this approach see [55–57]). This approach accounts for clustering in the data and standard errors are typically conservative [58]. See Additional file 2 (section 4) for details.

Good balance across all characteristics was achieved between synthetic comparison and original treatment groups (Additional file 2, section 4). However, there may still be fundamental differences between participated in the Trial and those who were selected for the synthetic control as there was considerable community involvement in the original Trial design to select the most vulnerable households. This required the deep understanding of the study population by community guides, which may not be captured by the data. Therefore, analyses of the original Trial data and with the synthetic comparison groups should be considered complimentary rather than substitutes.

#### Sub-group analyses

Sub-group analyses were conducted to evaluate differential effects of CTs by 1) type (UCTs and CCTs) and 2) sex of household head. Differential effects of CCT were expected given that the original evaluation of the Trial found slightly higher school attendance in the CCT group [35], which may impact outcomes (Fig. 3; Table 3). Analyses were implemented by sex of head of household as a possible indirect measure of the distribution of CTs within households, including possible gender-biased distribution.

## Results

### Primary outcomes

One thousand five hundred seventy individuals included in this analysis were aged 15–29 years (577 CCT, 536 UCT, and 457 control) and 1339 aged 30–54 years (448 CCT, 496 UCT, 395 control). Treatment and control groups were balanced across socio-demographic characteristics except for age and socio-economic status, with, on average, slightly higher socio-economic status in the control group. Therefore, all regressions adjusted for age and socio-economic status (determined in the Manicaland Cohort). See Additional file 2, section 3, for further information on the socio-demographic characteristics of the study sample. The same covariates were included in analyses with the synthetic comparison group for consistency, although good balance across all characteristics was achieved between synthetic comparison and original treatment groups (Additional file 2, section 3).

Compared to both the original and synthetic comparison group, receiving CTs had limited effect on sexual debut among those aged 15–20 years in the study population (Table 4). Among young people (15–29 years) (Table 4), living in households that received CTs reduced reporting of any sex in the past 30 days among males (56.6% [CT] vs. 71.9% [control]; ATE: -11.7PP [95% CI: -26.0PP, 2.61PP]) and females (69.9% vs 74.6%; ATE: -5.68PP [− 15.7PP, 4.34PP]). While uncertainty around these ATEs is large, estimates were similar with less uncertainty when compared against the synthetic comparison group (male ATE: -9.68PP [− 13.1PP, − 6.30PP]; female ATE: -8.77PP [− 16.3PP, − 1.23PP]) (Table 4). This effect was stronger in the CCT intervention among males (CCT ATE: -15.3PP [− 33.6PP, 3.04PP] vs. UCT ATE: -8.13PP [− 24.6PP, 8.39PP]) and females (CCT ATE: -6.63PP [− 18.0PP, 4.77PP]) vs. UCT ATE: -4.13PP [− 15.8PP, 7.55PP]) (see Additional file 3, section 2, for results by CT type, including with synthetic control groups). CTs had no effects on reporting any recent sexual intercourse among older individuals (30–54 years) in the sample (Table 5).

**Table 4:** Effects of cash transfers on young people (15–29 years) compared to the original and synthetic comparison groups, Manicaland Cash Transfer Trial, Manicaland, Zimbabwe, 2010–2011

Outcome:	Males (15–29)						Females (15–29)					
	Ctrl	CT	CT vs. ctrl (ref.)		CT vs. s. ctrl (ref.)		Ctrl	CT	CT vs. ctrl (ref.)		CT vs. s. ctrl (ref.)	
	n/N (%)	n/N (%)	ATE	(95% CI)	ATE	(95% CI)	n/N (%)	n/N (%)	ATE	(95% CI)	ATE	(95% CI)
**Primary outcomes**												
Had sexual debut^a^	7/156 (4.49)	28/391 (7.16)	1.43	(−3.18, 6.05)	−1.62	(−5.11, 1.87)	20/126 (15.9)	65/349 (18.6)	2.63	(−4.11, 9.38)	2.92	(−2.22, 8.05)
Had sex in past 30 days	41/57 (71.9)	69/122 (56.6)	−11.7	(−26.0, 2.61)	−9.68	(− 13.1, − 6.30)	82/110 (74.6)	197/282 (69.9)	−5.68	(− 15.7, 4.35)	− 8.77	(− 16.3, − 1.23)
Condom use (last sex)	21/57 (36.8)	51/122 (41.8)	2.68	(− 11.6, 17.0)	−2.49	(−9.70, 4.71)	19/110 (17.3)	46/282 (16.3)	−0.19	(−8.24, 7.86)	9.38	(5.90, 12.9)
Multiple partners^c^	10/57 (17.5)	31/122 (25.4)	8.49	(−5.40, 22.4)	10.3	(1.27, 19.2)	0/110 (0.00)	7/281 (2.49)	NA^b^		NA^b^	
**Secondary outcomes**												
Psychological distress^d^	29/232 (12.5)	58/535 (10.8)	−1.20	(−8.09, 5.69)	−3.00	(−6.75, 0.74)	52/225 (23.1)	116/578 (20.1)	−2.11	(−8.68, 4.46)	0.55	(−4.64, 5.74)
Enrolled in school^a^	101/156 (64.7)	281/391 (71.9)	11.5	(3.05, 19.9)	9.27	(3.05, 15.5)	82/126 (65.1)	227/349 (65.0)	−1.21	(−10.5, 8.07)	5.50	(1.62, 9.37)
Alcohol use^e^	34/232 (14.7)	64/535 (12.0)	−0.03	(−4.49, 4.43)	−4.03	(−8.49, 0.44)	0/225 (0.00)	2/578 (0.35)	NA^b^		NA^b^	
Smokes cigarettes	20/232 (8.62)	36/534 (6.74)	−0.38	(−4.06, 3.30)	−1.91	(−5.03, 1.21)	1/225 (0.44)	0/578 (0.00)	NA^b^		NA^b^	
Takes recreational drugs	26/232 (11.2)	47/533 (8.82)	−0.84	(−6.47, 4.78)	−2.91	(−6.78, 0.96)	1/225 (0.44)	1/578 (0.17)	NA^b^		NA^b^	

*Ctrl* Control, *CT* Cash transfer, *s. ctrl* Synthetic control, *ref.* Reference group, *ATE* Average treatment effect, *CI* Confidence interval

Sample: Young people (15–29 years) who had sex before for primary outcomes and all young people for secondary outcomes. The synthetic comparison group was determined through propensity score matching of individuals from the Manicaland Cohort to treatment-group individuals from the Manicaland Trial

Numbers are sample sizes of individuals reporting the outcome among everyone with data on the outcome (n/N) together with percentages (%) in the original control and treatment (CT) groups of the Manicaland Trial and estimated ATEs with 95% CIs when comparing the treatment (CT) groups against the original and synthetic comparison groups. ATEs for each outcome were estimated from separate mixed-effects logistic regression models, controlling for age and wealth index quarters (not shown), with study site and treatment cluster random effects. ATEs for comparisons against synthetic comparison groups were estimated from mixed-effects logistic regression models specified in the same way as for the original comparison group after propensity score matching. Propensity score matching for the control group was implemented with replacement. Full results, including p-values and sample sizes for synthetic comparison groups, can be found in Additional file 3 (section 1)

^a^ Analyses were restricted to those aged 15–20 years as it was not applicable to older individuals

^b^ No regression model was estimated due to sample size limitations

^c^ Having had more than one sexual partner in the past 12 months

^d^ Reporting at least 7 symptoms of psychological distress of a 25-item scale

^e^ Having been to a beer hall (bar) in the past month or drinking more than 3 drinks when drinking alcohol

**Table 5:** Effects of cash transfers on older people (30–54 years) compared to the original and synthetic comparison groups, Manicaland Cash Transfer Trial, Manicaland, Zimbabwe, 2010–2011

Outcome:	Males (30–54)						Females (30–54)					
	Ctrl	CT	CT vs. ctrl (ref.)		CT vs. s. ctrl (ref.)		Ctrl	CT	CT vs. ctrl (ref.)		CT vs. s. ctrl (ref.)	
	n/N (%)	n/N (%)	ATE	(95% CI)	ATE	(95% CI)	n/N (%)	n/N (%)	ATE	(95% CI)	ATE	(95% CI)
**Primary outcomes**												
Had sex in past 30 days	84/92 (91.3)	207/239 (86.6)	−5.05	(−12.4, 2.25)	−0.25	(− 8.56, 8.05)	152/300 (50.7)	334/696 (48.0)	−1.61	(−8.29, 5.07)	−3.04	(− 10.3, 4.24)
Condom use (last sex)	22/92 (23.9)	48/239 (20.1)	−2.30	(−12.7, 8.08)	7.64	(−0.57, 15.8)	67/300 (22.3)	160/695 (23.0)	1.16	(−5.09, 7.42)	5.95	(1.46, 10.4)
Multiple partners^a^	13/92 (14.1)	24/240 (10.0)	−2.84	(−10.9, 5.26)	−2.54	(−8.82, 3.73)	5/300 (1.67)	12/695 (1.73)	−0.40	(−1.71, 0.91)	0.02	(−0.44, 0.47)
**Secondary outcomes**												
Psychological distress^b^	16/94 (17.0)	41/243 (16.9)	− 4.42	(− 15.7, 6.82)	1.27	(−5.33, 7.88)	118/301 (39.2)	254/701 (36.2)	−3.97	(− 11.7, 3.75)	3.00	(−1.62, 7.63)
Alcohol use^c^	38/94 (40.4)	104/243 (42.8)	3.57	(−11.2, 18.3)	−10.7	(−22.7, 1.22)	2/301 (0.66)	9/700 (1.29)	NA^d^		NA^d^	
Smokes cigarettes	32/94 (34.0)	80/243 (32.9)	−2.06	(−14.8, 10.7)	−4.05	(−16.4, 8.27)	2/300 (0.67)	5/700 (0.71)	NA^d^		NA^d^	
Takes recreational drugs	35/94 (37.2)	63/242 (26.0)	−10.5	(−23.5, 2.59)	−4.80	(−15.1, 5.53)	1/301 (0.33)	8/697 (1.15)	NA^d^		NA^d^	

*Ctrl* Control, *CT* Cash transfer, *s. ctrl* Synthetic control, *ref.* Reference group, *ATE* Average treatment effect, *CI* Confidence interval

Sample: Older people (30–54 years) who had sex before for primary outcomes and all young people for secondary outcomes. The synthetic comparison group was determined through propensity score matching of individuals from the Manicaland Cohort to treatment-group individuals from the Manicaland Trial

Numbers are sample sizes of individuals reporting the outcome among everyone with data on the outcome (n/N) together with percentages (%) in the original control and treatment (CT) groups of the Manicaland Trial and estimated ATEs with 95% CIs when comparing the treatment (CT) groups against the original and synthetic comparison groups. ATEs for each outcome were estimated from separate mixed-effects logistic regression models, controlling for age and wealth index quarters (not shown), with study site and treatment cluster random effects. ATEs for comparisons against synthetic comparison groups were estimated from mixed-effects logistic regression models specified in the same way as for the original comparison group after propensity score matching. Propensity score matching for the control group was implemented with replacement. Full results, including sample sizes for synthetic comparison groups, can be found in Additional file 3 (section 1).

^a^ Having had more than one sexual partner in the past 12 months

^b^ Reporting at least 7 symptoms of psychological distress of a 25-item scale

^c^ Having been to a beer hall (bar) in the past month or drinking more than 3 drinks when drinking alcohol

^d^ No regression model was estimated due to sample size limitations

Compared to the original control group, condom use was not affected by receiving CTs among young (Table 4) and older people (Table 5). Analyses with the synthetic comparison group suggest that condom use was increased by 9.38PP (5.90PP, 12.9PP) among young females but not among young males (ATE: − 2.49 [− 9.70, 4.71] (Table 4) as well as by 5.95PP (1.46PP, 10.4PP) among older females and by 7.64PP (− 0.57PP, 15.8PP) among older males (Table 5).

Young (but not older) males receiving CTs were more likely to report having more than one sexual partner in the past 12 months (25.4%) compared to those not receiving CTs (17.5%) (ATE: 8.49PP [− 5.40PP, 22.4PP]) (Table 4). The effect was similar with less uncertainty when compared to the synthetic comparison group (ATE: 10.3PP [1.27PP, 19.2PP]) and similar in the UCT and CCT interventions (Additional file 3, section 2). Few females reported multiple sexual partners and there were no effects (Tables 4 and 5).

### Secondary outcomes

**Fig. 4:**
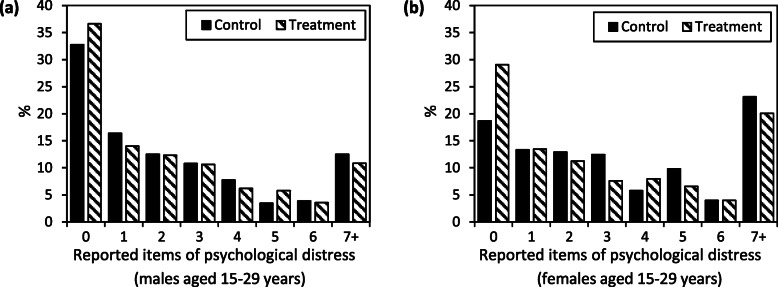
Psychological distress among young people, Manicaland Cash Transfer Trial, Manicaland, Zimbabwe, 2010–2011. Number of positive responses to questions on psychological distress by young males (**a**) and females (**b**) (aged 15–29 years) in the control and treatment (cash transfer) groups

11.3% of young males and 20.9% of young females were psychologically distressed (reporting at least seven of 25 items of the psychological distress scale) (Fig. 4). There were only small or no changes in proportions classified as psychologically distressed among young people receiving CTs (Table 4). However, there was an overall shift in the distribution of the number of reported symptoms of psychological distress, with higher proportions of young males and females reporting no symptoms in the CT group (Fig. 4). Reductions in psychological distress among older individuals in households receiving CTs were small (Table 5).

In the study population, CTs led to increased school enrolment among young males (15–20 years) (71.9% vs 64.7%; ATE: 11.5PP [3.05PP, 19.9PP]), with a similar effect when compared to the synthetic comparison group (ATE: 9.27PP [3.05PP, 15.5PP]) (Table 4). Among young females, there was no effect on school enrolment among those receiving CTs (65.1%) when compared to the original control group (65.0%) (ATE: -1.21PP [− 10.5PP, 8.07PP]) but an increase compared to the synthetic comparison group (ATE: 5.50PP [1.62PP, 9.37PP]) (Table 4). The effect was slightly stronger among males in the CCT intervention (ATE: 13.0PP [5.19PP, 20.7PP] vs. UCT ATE: 10.9PP [1.28PP, 20.5PP]), which was also found in analyses of the synthetic comparison group (Additional file 3, section 2). School enrolment was hypothesised to be an important mediating factor for the effects of CTs on sexual behaviour (Fig. 3; Table 3) and sexual activity among those enrolled in school was low. About 2% of young people aged 15–20 years enrolled in school had sex before, with slightly higher proportions for those in households receiving CTs (males: 2.49% [7/281] vs. 1.98% [2/101] in the control group; females: 2.64% [6/227] vs. 0% [0/82]) (not shown).

Among young males receiving CTs, no increases in alcohol, cigarette, or recreational drug consumption was observed, with lower alcohol use compared to the synthetic comparison group (11.6% vs. 15.7%; ATE: -4.03PP [− 8.49PP, 0.44PP]) (Table 4). Negative and weak effects were found among older males (except for a positive but weak association with alcohol use, which is strongly negative when compared to the synthetic comparison group; Table 5). Few females reported alcohol, cigarettes, or drug consumption (Tables 4 and 5).

### Effects in male- and female-headed households

Roughly equal proportions of young males and females lived in male- and female-headed households, while 88.1% of older men and 40.9% of older females lived in male-headed households. Few young people were themselves heads of households (6.36% of males; 5.02% of females) while most older men (83.5%) and half of older women (52.1%) were heads of households. See Additional file 3 (section 3) for details.

In the study population, even though uncertainty is large, results suggests that CTs reduced having any recent sexual activity more strongly among young males living in households headed by a male (59.7% vs. 85.3%; ATE: -20.0PP [− 39.1PP, − 0.90PP]) compared to young males living in female-headed households (52.7% vs. 52.2%; ATE: 1.29PP [− 21.7PP, 24.3PP]), while CTs increased multiple partnerships regardless of type of household (Table 6). Among young males aged 15–20 years, CTs increased school enrolment more strongly in male-headed households (71.3% vs. 56.7%; ATE: 17.9PP [6.65PP, 29.1PP]) than in female-headed households (72.4% vs. 69.8%; ATE: 8.28PP [− 2.68PP, 19.2PP]) (Table 6) – even when excluding young males who themselves were heads of households (ATE in male-headed households: 17.2PP [5.40PP, 29.02PP]) (not shown). Young males in female-headed households were generally more likely to be enrolled in school, with 69.8% of young males in control households headed by females enrolled in school compared to 56.7% in male-headed control households.

**Table 6:** Effects of cash transfers on young people (15–29 years) in male- and female-headed households compared to the original comparison group, Manicaland Cash Transfer Trial, Manicaland, Zimbabwe, 2010–2011

Outcome:	Males (15–29)								Females (15–29)							
	Male-headed households				Female-headed households				Male-headed households				Female-headed households			
	%		CT vs. ctrl (ref.)		%		CT vs. ctrl (ref.)		%		CT vs. ctrl (ref.)		%		CT vs. ctrl (ref.)	
	Ctrl	CT	ATE	(95% CI)	Ctrl	CT	ATE	(95% CI)	Ctrl	CT	ATE	(95% CI)	Ctrl	CT	ATE	(95% CI)
**Primary outcomes**																
Had sexual debut^a^	3.33	8.62	5.18	(−0.59, 10.9)	5.21	5.99	−1.58	(−8.55, 5.38)	19.3	21.1	4.56	(−5.58, 14.7)	13.0	16.8	−0.09	(−9.45, 9.27)
Had sex in past 30 days	85.3	59.7	−20.0	(−39.1, −0.90)	52.2	52.7	1.29	(−21.7, 24.3)	85.9	79.4	−7.57	(−18.1, 2.92)	53.9	52.5	2.51	(−16.6, 21.6)
Condom use (last sex)	26.5	40.3	10.9	(−8.41, 30.1)	52.2	43.6	−12.9	(−35.5, 9.76)	12.7	13.9	1.74	(−7.40, 10.9)	25.6	20.8	−2.52	(−20.6, 15.5)
Multiple partners^c^	20.6	28.4	3.30	(−16.5, 23.1)	13.0	21.8	6.92	(−11.9, 25.7)	0.00	2.78	NA^b^		0.00	2.00	NA^b^	
**Secondary outcomes**																
Psychological distress^d^	11.7	12.8	1.27	(−6.28, 8.82)	13.3	9.12	−4.55	(−12.8, 3.72)	27.5	23.3	−3.87	(− 12.9, 5.20)	18.1	16.7	0.34	(−8.18, 8.86)
Enrolled in school^a^	56.7	71.3	17.9	(6.65, 29.1)	69.8	72.4	8.28	(−2.68, 19.2)	63.2	58.6	−8.94	(−20.1, 2.19)	66.7	69.9	5.15	(−6.48, 16.8)
Alcohol use^e^	20.4	14.0	0.60	(−6.70, 7.91)	10.2	10.2	−1.17	(−6.89, 4.55)	0.00	0.00	NA^b^		0.00	0.73	NA^b^	
Smokes cigarettes	10.7	7.20	0.07	(−5.69, 5.83)	7.03	6.34	−2.48	(−7.70, 2.75)	0.83	0.00	NA^b^		0.00	0.00	NA^b^	
Takes recreational drugs	15.5	8.80	−3.50	(−12.1, 5.05)	7.81	8.83	−0.18	(−6.23, 5.86)	0.83	0.33	NA^b^		0.00	0.00	NA^b^	

*Ctrl* Control, *CT* Cash transfer, *ref.* Reference group, *ATE* Average treatment effect, *CI* Confidence interval

Sample: Young people (15–29 years) who had sex before for primary outcomes and all young people for secondary outcomes, restricted to male- and female-headed households, respectively

Numbers are percentages (%) of individuals reporting the outcome in the control and treatment (CT) groups of the Manicaland Trial and the estimated ATEs with 95% CIs, separately for individuals living in male- and female-headed households. ATEs for each outcome were estimated from separate mixed-effects logistic regression model, controlling for age and wealth index quarters (not shown), with study site and treatment cluster random effects. No results for transactional sex are shown because of very small sample sizes. Full results, including *p*-values and sample sizes, can be found in Additional file 3 (section 2)

^a^ Analyses were restricted to those aged 15–20 years as it was not applicable to older individuals

^b^ No regression model was estimated due to sample size limitations

^c^ Having had more than one sexual partner in the past 12 months

^d^ Reporting at least 7 symptoms of psychological distress of a 25-item scale

^e^ Having been to a beer hall (bar) in the past month or drinking more than 3 drinks when drinking alcohol

Young females tended to be less likely to report any recent sexual activity in male-headed households receiving CTs (79.4% vs. 85.9%; ATE: -7.57PP [− 18.1PP, 2.92PP]) but not in female-headed households (52.5% vs. 53.9%; ATE: 2.51PP [− 16.6PP, 21.6PP]), although control-group females were generally less likely to report any recent sexual activity when living in female-headed households (53.9%) compared to male-headed households (85.9%) (Table 6). CTs did not increase school enrolment among females (15–20 years) in male-headed households (63.2% vs. 58.6%; ATE: -8.94PP [− 20.1PP, 2.19PP]) but did so in in female-headed households (69.9% vs. 66.7%; ATE: 5.15PP [− 6.48PP, 16.8PP]) (Table 6). Effects on school enrolment were similar when excluding younger females who were heads of households (ATE in female-headed households: 4.18PP [− 7.91PP, 16.27PP]) (not shown).

Among older females, there were considerable differences in reporting of any recent sexual activity between control households headed by a male (85.0%) and those headed by a female (30.0%). However, CTs had similar effects on any recent sexual activity in male- (80.9% vs. 85.0%; ATE: -4.28PP [− 12.2PP, 3.60PP]) and female-headed households (24.9% vs. 30.0%; ATE: -3.94PP [− 11.6PP, 3.71PP]). Uncertainty around estimates for older males in female-headed households was high given small sample sizes. See Additional file 3 (section 3) for results on older individuals.

## Discussion

This study of largely rural communities in eastern Zimbabwe affected by a generalised HIV epidemic found evidence that a CT intervention without HIV/STI-specific objectives had spillover effects relevant for HIV/STI prevention. These included lower recent sexual activity among young people, possibly increased condom use, and increased multiple partnerships among young males, while there were no effects on sexual debut or effects on recent sexual activity among older people. Especially among young men, school enrolment was increased. There was no increased alcohol, cigarette, or drug consumption in households receiving CTs.

### CTs and sexual behaviour

This study found no effects on sexual debut among males and females aged 15–20 years, while the HSCT programme evaluation in Zimbabwe found a 13PP reduction in sexual debut among those aged 13–20 years after 12-month [36] (−9PP after 48 months [25]). In the Manicaland Trial, levels of sexual debut were low (4.49% among control males; 15.9% among females), compared to 17% in the treatment and 28% in the control group after 12 months in the HSCT evaluation [36]. Similarly, other CT programmes that reduced sexual debut (in Kenya [13] and Malawi [12]) had higher levels of sexual debut compared to Manicaland. Therefore, reductions in sexual debut due to CTs may depend on baseline levels of sexual debut. On the other hand, this study found reductions in any recent sexual activity among sexually active young people (15–29 years), similar to other CT interventions [11, 12, 17, 18]. The HSCT evaluation found no effect on numbers of sex acts in the past 3 months [25, 36], but measures may not be comparable.

Young males living in CT-receiving households were more likely to report multiple partners in the past year. The HSCT evaluation found that, after 48 months, males (13–20 years) in CT-receiving households had, on average, 1.67 sexual partners in the past 12 months compared to 1.23 in control households [25]. CT evaluations in Tanzania [21] and Malawi [12] found no effects on multiple partnerships among young males while reductions were found in Kenya [13, 14] and South Africa [10], although multiple partnerships were more common in South Africa (30% among those aged 15–17 years vs. 18% in the Manicaland young male control group). In the Manicaland Trial, CT could have led to economic empowerment of young men to attract more sexual partners, but reasons for the increase of multiple partnerships are unclear. However, it underscores the importance of monitoring possible adverse effects of CTs.

Compared to the synthetic comparison group, CTs in the Manicaland Trial were found to increase condom use during last sex among younger and older women and among older men. Similarly, significant reductions in unprotected sex in the past 3 months among CT recipients were found among young males and females after 48 months in the HSCT evaluation [25] and schoolgirls in the HPTN 068 trial [16], while other CT evaluations did not find effects on condom use [10, 12, 13]. Again, these contrasting effects could be the result of different baseline levels in condom use.

### CTs and school enrolment

The 10PP increase in school enrolment among young males in households receiving CTs was similarly found CT evaluations in Kenya [27] and Malawi [12]. Similar to the current study, the study in Kenya did not find effects on school enrolment among females [27], while the evaluation in Malawi similar effects among males and females. In the SIHR study, CT-receiving schoolgirls enrolled in school at baseline were more likely to be still enrolled in school at follow-up [18], with CCTs having stronger effects, similar to findings of the current study and the original Manicaland Trial evaluation [35]. A previous analysis of the Manicaland Trial found that CCTs were particularly effective at replacing child labour with school attendance [59]. High acceptance of the conditions in the CCT group was found in qualitative studies of the Trial [60, 61], which were seen as a way to promote good parenting and build social accountability, possibly explaining stronger effects of the CCT intervention.

The HSCT evaluation in Zimbabwe did not find effects of CTs on primary or secondary school enrolment or grade progression after 12 or 48 months [25, 36], with similar levels of baseline school enrolment as in the Manicaland Study. The HSCT programme was found to have negative impacts on receiving secondary school scholarships under the Basic Education Assistance Module (BEAM) [25], thus offsetting any positive effects of receiving CTs. Differences between the Manicaland Trial and HSCT programme illustrate that large-scale programmes may not have the same effects as trials which are implemented under more controlled conditions. Specifically, coordination was lacking between the separate ministries implementing the HSCT and BEAM programmes [25].

Increased school enrolment due to CTs may partially explain lower reporting of any recent sexual activity among young people. School enrolment was more strongly increased among males and among those receiving CCTs, among which reductions in sexual activity was stronger. Although studies tend to focus on females, school attendance has been shown to reduce HIV risks through delayed sexual debut and changes in sexual behaviour [44, 45], and a Kenyan CT programme evaluation found school enrolment to mediate changing sexual behaviour [27]. Mediating effects of school enrolment may also explain why fewer and weaker effects of CTs were observed among older people (who may also be more likely to be in more stable sexual relationships). The short Trial duration and focus on improving school enrolment may have not generated a sufficient income effect that could have impacted outcomes among older people. However, given that young females in households receiving CTs in the Trial showed reductions in any recent sexual activity and increased condom use while effects on school enrolment were limited, other factors are likely to mediate between CTs and sexual behaviour, which has been similarly found in Kenya [27] and South Africa [16].

### CTs and mental health

The Manicaland Trial did not find effects of CTs on proportions of males or females classified as psychologically distressed, similar to evaluations of the HSCT programme in Zimbabwe which found no effects on proportions classified as depressed [25, 36]. Nevertheless, there were indications that CTs in Manicaland shifted distributions of reported symptoms of psychological distress and a qualitative study of the Trial found that children and guardians in households receiving CTs reported reduced levels of stress and anxiety [61]. A previous analysis of the study population found links between psychological distress and potentially risky sexual behaviour [46], so improved mental health may have contributed to reduced recent sexual activity, although reverse causality is possible. Similarly, the Kenyan CT evaluation found a mediating role of mental health [27]. Among schoolgirls in a CT programme in Malawi, which split varying amounts of CTs between schoolgirls and guardians, psychological distress was reduced by 14PP by UCTs and 6PP by CCTs [62]. Limited improvement in mental health in the CCT group was largely due to increased distress among schoolgirls whose parents received larger cash amounts, suggesting that CTs can cause distress when they become an important source of household income and depend on the adolescents’ behaviour. Increasing psychological distress among some individuals may contribute to lack of average effects identified in this study, although similarly weak effects were found in the UCT and CCT interventions.

### Alcohol, cigarette, and drug consumption

Similar to evaluations of the HSCT programme in Zimbabwe as well as the HPTN 068 trial and national programmes in South Africa [10, 14, 28], this study did not find evidence that individuals living in CT households increased alcohol, cigarette, or drug consumption; in fact, these behaviours may be reduced by CTs, which was also indicated by the HSCT evaluation [25]. While qualitative studies of the Trial reported that older men were considered to misappropriate CTs [60, 61], including for alcohol, this study indicates that, if anything, this did not occur on a large scale.

### Effects by sex of head of household

Given that CTs were distributed to household heads in the Manicaland Trial, the distribution of cash within households likely influenced whether individuals’ behaviours changed, and this study found different effects of CTs in households headed by males or females. CT increased school enrolment among males more strongly in male-headed households, and female school enrolment was only increased in female-headed households. Given that caring for an orphan was one Trial eligibility criteria, a large proportion of young people included in the analysis were orphans (both parents were alive for about one-third of those aged under 18). Previous mixed-methods studies in the study population found that children whose fathers died were more likely to live in female-headed households with the mother or other female relatives while children whose mothers died were less likely to live with the surviving father [63]. Moreover, living with the surviving mother or another female relative was found to be beneficial for primary school completion, particularly for girls, possibly due to reduced gender bias in resource allocation in households. Living with surviving fathers had detrimental effects on maternal orphans’ school completion, particularly for girls. This may explain why there was no increased school enrolment due to CTs among females living in male-headed households and why there was an effect on males in male- and female-headed households but this was stronger in male-headed households. This advantage of living in female-headed households is also reflected in generally higher levels of school enrolment and the gender bias in resource allocation is reflected in higher male school enrolment regardless of household type. This indicates that household characteristics are important as to whether CTs have effects. This is further illustrated by effects on any recent sexual activity, which was reduced among young males and females only in male-headed households receiving CTs, which had higher baseline sexual activity levels for both sexes, possibly because orphans living in male-headed households are less likely to live with the father [63].

### Limitations

Limited sample size resulted in high uncertainty around estimates, particularly in sub-analyses. Effective sample sizes and statistical power to detect effects of CTs may have been further reduced by the fact that the Manicaland Cohort data were collected over 20 months, so some study sites were covered early during the Manicaland Trial and study participants may have not received a large enough CT ‘dose’ for measurable impacts on outcomes. However, analyses of the synthetic comparison groups, which had larger sample sizes, largely confirmed results of analyses of the original Trial sample, although there may be fundamental differences between those who participated in the Trial and those who did not (who became part of the synthetic comparison group) – despite good balance of the original treatment and synthetic comparison groups in terms of socio-demographic and economic characteristics. These differences may not be captured in the data, particularly as community groups guided the selection of the most disadvantaged households. Therefore, results that differed markedly between original and synthetic comparison group analyses, including for condom use, need to be considered with caution. The fact that the distribution of CTs within households was unknown likely further introduced imprecision in estimates as individuals may have been included in analyses that did not benefitted from CTs given to households. This means that CT effects that were identified in the study are likely to be underestimates. Additionally, with analyses of the original and synthetic control groups, a large number of statistical tests have been conducted, so some apparent effects may have been found by chance. Nevertheless, there is a general consistency between results and prior hypotheses, often supported by results across several comparisons, providing confidence into the conclusions drawn from the analyses.

A further limitation was that data on measures were taken from the Manicaland Cohort that were not designed for analysing the Trial. For example, the survey question for multiple partnerships asked about the number of partners in the past 12 months, which may include a period before the Trial started. However, while this may make it more difficult to identify effects of CTs (committing type II statistical errors), these limitations apply equally to treatment and control groups, thus CT effects identified in this study are unlikely to be biased. Moreover, this study relied on potentially biased self-reporting – despite using informal confidential voting methods to reduce social desirability bias [64] – but these biases are unlikely to differ between Trial groups, so differences between groups is unlikely the result of differential reporting. The design of this analysis is also likely to have reduced reporting bias as outcome data was collected in the Manicaland Cohort independently from the Manicaland Trial, unlike other CT trials that tend to be unblinded and thus more prone to reporting bias.

As only one measurement of each outcome was available from the Manicaland Cohort data, baseline balance between Trial groups could not be analysed. However, there was a good balance between groups in terms of characteristics unlikely to change within short time periods and analyses controlled for age and socio-economic status. Lack of baseline measures further prevented analyses of temporal changes in study outcomes in control areas. Individuals in Trial control households received fertilisers and seeds, so a general socio-economic improvement may have made it more difficult to identify differences between Trial groups. The Trial was also implemented during a period of economic recovery, which has been found to have affected sexual behaviour of the study population, including increases in multiple partnerships [65]. However, these broader economic trends impacted treatment and control groups and are unlikely to bias comparisons between groups.

Selection bias when combining the Trial and Cohort data could have been introduced if there were differences between Trial participants linked to the Cohort and those not linked, and these patterns differed between treatment and control groups. However, systematic differences between those included in this analysis and those not were similar between Trial groups, suggesting no bias was introduced at this stage. The systematic differences between those linked to the Cohort and those not may be because, for the baseline Trial survey, heads of households provided data for everyone living in households. This may have inflated numbers of young people listed as living in the households compared to numbers of people found during the Cohort survey, leading to artificial differences in age structures.

## Conclusions

This study, using an innovative evaluation approach of combining data sources, demonstrates that a CT intervention aiming to increase educational and health outcomes among children had spillover effects relevant for HIV/STI prevention. These include positive externalities further supporting CT as a social protection policy, including increased school enrolment, reduced sexual activity, and more condom use, and results are likely to be generalisable to other CT interventions in rural sub-Saharan Africa that target socio-economically disadvantaged households, particularly as the Trial involved different study site types [66]. Nevertheless, effects of CTs may depend on context and baseline levels, e.g. of school enrolment, and Trial results may not be replicated in large national programmes. CT programmes are costly but the diversity in outcomes of structural interventions is often not captured in economic evaluations [67]. Economic evaluation modelling suggests that considering only HIV infections averted as the outcome in cost-effectiveness analyses may lead to suboptimal resource allocation decisions compared to cost-benefit analyses accounting for other health and educational outcomes [68]. Such a cross-sector evaluations of CT programmes, as promoted by UNICEF [69], is also required to detect negative externalities such as increased multiple sexual partnerships among young men living in CT-receiving households in the Manicaland Trial. This and other results of this study demonstrate that CTs may have differential effects on different population groups. Gender-specific effects are not commonly considered and effects of CTs on males are less well-understood than for females. Different effects by type of households found in this study suggest possibilities to target CTs to specific households. For example, female-headed households may be targeted to maximise effects on young women. Questions remain regarding the pathways through which CTs influence behaviour and the sustainability of the effects, but this study adds to the growing body of literature that suggest that CT can have a broad range of beneficial effects for development, addressing structural drivers of HIV infection.

## Supplementary information

**Additional file 1.** Contains more details on the literature review on CTs and HIV/STI prevention.

**Additional file 2.** Contains the following sections, referred to throughout the article: 1. Additional information on data and measures (p.2). 2. Characteristics of the analysed sample compared to those not included (p.5). 3. Socio-demographic characteristics and balance of Trial groups (p.6). 4. Additional information on methods and results from propensity score matching (p.8)

**Additional file 3 **Contains the following sections, referred to throughout the article: 1. Sample sizes and *p*-values for main results tables (p.2). 2. Results by type of CT intervention (CCT and UCT) (p.7). 3. Additional results by type of sex of head of household (p.12)

## Data Availability

Data produced by the Manicaland Project, including from the Manicaland General-Population Cohort, can be obtained from the project website: http://www.manicalandhivproject.org/data.html. Here we provide a core dataset which contains a sample of socio-demographic, sexual behaviour and HIV testing variables from all 6 surveys of the main survey. If further data is required, a data request form must be completed (available to download from our website) and submitted to simon.gregson@imperial.ac.uk.

## Abbreviations

AIDS: Acquired immunodeficiency syndrome
ATE: Average treatment effect
BEAM: Basic Education Assistance Module
CI: Confidence interval
CSG: Child Support Grant programme (in South Africa)
CT: Cash transfer
CCT: Conditional cash transfer
UCT: Unconditional cash transfer
CT-OVC: Cash Transfer for Orphans and Vulnerable Children programme (in Kenya)
GEE: Generalised estimating eq.
HIV: Human immunodeficiency virus
HPTN: HIV prevention trial network
HSCT: Harmonised Social Cash Transfer programme (in Zimbabwe)
IPV: Intimate partner violence
MPSLSW: Ministry of Public Service, Labour and Social Welfare (in Zimbabwe)
NSP: National social protection programme (in South Africa)
aOR: Adjusted odds ratio
OVC: Orphans and vulnerable children
PP: Percentage point
PrEP: Pre-exposure prophylaxis
PS: Propensity score
PSM: Propensity score matching
RCT: Randomised controlled trial
cRCT: Cluster-randomised controlled trial
SCTP: Social Cash Transfer Programme (in Malawi)
SIHR: Schooling, Income, and Health Risk study (in Malawi)
STI: Sexually transmitted infection
ZOE: Zimbabwe Orphan Endeavor programme (in Kenya)
