# Supplementary material for: Spillover HIV prevention effects of a cash transfer trial in East Zimbabwe: evidence from a cluster-randomised trial and general-population survey
Source: BMC Public Health. 2020 Oct 23;20:1599. doi: 10.1186/s12889-020-09667-5 (PMC7584095; doi:10.1186/s12889-020-09667-5)
Supplement: Supplementary file 1 — Additional file 1. Contains more details on the literature review on CTs and HIV/STI prevention. [file 12889_2020_9667_MOESM1_ESM.docx]

**Additional file 1**

**Spillover HIV prevention effects of a cash transfer trial on child development in East Zimbabwe: Evidence from a cluster-randomised trial and general-population survey**

**Schaefer et al.**

**BMC Public Health**

**Introduction**

This supplementary material contains a more extensive review of the literature on CTs and HIV/STI prevention to supplement the information provided in the main article.

## Review of evidence of CTs for HIV prevention

Benefits of CTs for HIV/STI prevention have been examined in randomised controlled trials (RCTs) [1, 2] and evaluations of national CT programmes [3-7], as summarised in Tables 1-2. In South Africa, the HPTN 068 study found no effect of conditional CTs given to young women (13-20 years) to promote school attendance on HIV or herpes simplex virus type 2 (HSV-2) incidence in this population [8], although CTs led to a reduction in intimate partner violence (IPV) and number of sexual partners and delayed sexual debut [9]. In contrast, the CAPRISA 007 trial in South Africa found a significant reduction in HSV-2 incidence among male and female high-school students receiving CTs conditional on meeting at least one of several conditions (80% participation in a life skills programme, attaining passing scores in academic tests, and annual HIV testing). [10] In Malawi, the Schooling, Income, and Health Risk (SIHR) study found reduced HIV and HSV-2 prevalence among young women (13-22 years) receiving UCTs and CCTs in addition to delayed sexual debut, fewer sexual partners, and smaller age differences to partners [11], although effects on partner age differences were weak [12]. However, most CT effects in the SIHR study disappeared after the end of the trial. [13] The Malawi Incentives Project involved CTs conditional on staying HIV-negative and no effects of CTs were observed on any measure of sexual behaviour [14]; however, after receiving CTs at the end of the trial, males reported a net increase in potentially risky sexual behaviour (more condom use but also more sex) while females reported a net decrease (no effect on condom use but less sex). Another RCT with CTs conditional on staying STI-free in Lesotho showed a reduction in HIV incidence and prevalence and in STI prevalence among those in the intervention arm of the trial [15], but HIV incidence was similar between intervention and control groups one year after the trial ended. The RESPECT Study in Tanzania was a similar trial of CCTs that showed a reduction in STI prevalence among both sexes (18-30 years) after 12 months [16], although this effect was only sustained among males in a one-year follow-up survey [17].

In addition to purposively designed trials, national CT programmes have been evaluated in terms of spillover effects relevant for HIV/STI prevention. In South Africa, the national social protection (NSP) programme, involving monthly UCTs given to caregivers of foster children, has been found to decrease transactional sex and age difference with sexual partners among girls (10-18 years) and multiple partnerships among boys (10-18). [4] Similarly, the South African Child Support Grant (CSG) programme, giving UCTs to caregivers of children in low-income households, has been found to be associated with delayed sexual debut among females but not males (15-16 years). [5] The Cash Transfer for Orphans and Vulnerable Children (CT-OVC) programme in Kenya has been found to be associated with delayed sexual debut among both sexes (15-25 years) [3] and reduced early marriage and pregnancy among females [18]. Another intervention in Kenya, referred to as the Zimbabwe Orphan Endeavor (ZOE) programme, combined UCTs given to young males and females who had household caring responsibilities with psychosocial support and entrepreneurship training and has been found to be associated with delayed sexual debut. [6] In Malawi, an evaluation the Social Cash Transfer Programme (SCTP), involving monthly UCTs for the poorest households, found that young males (13-19 years) living in households receiving CTs were less likely to have had sex before and young females had more age-similar partners at first sex and were less likely to report that their first sex was forced. [7]

These evaluations of CTs in trials or national programmes demonstrate that CTs can have effects on sexual behaviour that are associated with reduced risks for HIV/STI infection (delayed sexual debut, fewer and more age-similar partners, and condom use), although few studies demonstrate effects on biological outcomes. There are further questions regarding the sustainability of the effects of CTs on behaviour and causal pathways of these effects. [19] Trials on CTs tended to focus on increasing school enrolment [8, 10, 11], while one study of the impact of the Kenyan UCT programme indicated that observed effects were only partially mediated by increased school enrolment [20]. A major limitation of studies of CTs is the focus on younger people, particularly young females, with effects among males being less clear. This is particularly relevant in the context of potential adverse effects of CTs in terms of possibly health-damaging behaviours, including risky sex and alcohol, cigarette, and drug consumption, which may be more common among males. Such effects are not commonly evaluated and reported. A mixed-methods study on the impact of the HPTN 068 trial found that there was only limited spending of CTs on alcohol, cigarettes, or drugs and no harassment by partners was reported. [21] Similarly, two evaluations of South African national CT programmes found no indications for adverse effects. [4, 5] In contrast, the evaluation of the ZOE Orphan Empowerment found indications for increased unprotected sex among males receiving CTs. [6] In a qualitative study of a pilot CT programme in Johannesburg, participants reported that spending on drugs and alcohol was common among CT recipients and that some males engaged in criminal activities after the trial ended to compensate for the reduced income. [22] In the MIP, males reported more condomless sex after receiving CCTs. [14]

**References**

1. Pettifor A, MacPhail C, Nguyen N, Rosenberg M: **Can money prevent the spread of HIV? A review of cash payments for HIV prevention**. *AIDS and Behavior* 2012, **16**(7):1729-1738.

2. Taaffe J, Cheikh N, Wilson D: **The use of cash transfers for HIV prevention — are we there yet?** *African Journal of AIDS Research* 2016, **15**(1):17-25.

3. Handa S, Halpern CT, Pettifor A, Thirumurthy H: **The Government of Kenya's Cash Transfer Program Reduces the Risk of Sexual Debut among Young People Age 15-25**. *PLOS ONE* 2014, **9**(1):e85473.

4. Cluver L, Boyes M, Orkin M, Pantelic M, Molwena T, Sherr L: **Child-focused state cash transfers and adolescent risk of HIV infection in South Africa: a propensity-score-matched case-control study**. *The Lancet Global Health* 2013, **1**(6):e362-370.

5. Heinrich CJ, Hoddinott J, Samson M: **Reducing Adolescent Risky Behaviors in a High-Risk Context: The Effects of Unconditional Cash Transfers in South Africa**. *Economic Development and Cultural Change* 2017, **65**(4):619-652.

6. Goodman ML, Selwyn BJ, Morgan RO, Lloyd LE, Mwongera M, Gitari S, Keiser PH: **Sexual Behavior Among Young Carers in the Context of a Kenyan Empowerment Program Combining Cash-Transfer, Psychosocial Support, and Entrepreneurship**. *Journal of sex research* 2016, **53**(3):331-345.

7. Malawi Social Cash Transfer Programme (SCTP) Evaluation Team: **Malawi Social Cash Transfer Programme: Endline Impact Evaluation Report**. In*.* Chapel Hill: Carolina Population Center; 2016.

8. Pettifor A, MacPhail C, Hughes JP, Selin A, Wang J, Gomez-Olive FX, Eshleman SH, Wagner RG, Mabuza W, Khoza N *et al*: **The effect of a conditional cash transfer on HIV incidence in young women in rural South Africa (HPTN 068): a phase 3, randomised controlled trial**. *Lancet Glob Health* 2016, **4**(12):e978-e988.

9. Kilburn KN, Pettifor A, Edwards JK, Selin A, Twine R, MacPhail C, Wagner R, Hughes JP, Wang J, Kahn K: **Conditional cash transfers and the reduction in partner violence for young women: an investigation of causal pathways using evidence from a randomized experiment in South Africa (HPTN 068)**. *J Int AIDS Soc* 2018, **21 Suppl 1**.

10. Abdool Karim Q, Leask K, Kharsany A, Humphries H, Ntombela F, Samsunder N, Baxter C, Frohlich J, van der Elst L, Abdool Karim SS: **Impact of conditional cash incentives on HSV-2 and HIV prevention in rural South African high school students: results of CAPRISA 007 cluster randomized trial**. In: *International AIDS Conference.* Vancouver, Canada; 2015.

11. Baird SJ, Garfein RS, McIntosh CT, Ozler B: **Effect of a cash transfer programme for schooling on prevalence of HIV and herpes simplex type 2 in Malawi: a cluster randomised trial**. *Lancet (London, England)* 2012, **379**(9823):1320-1329.

12. Beauclair R, Dushoff J, Delva W: **Partner age differences and associated sexual risk behaviours among adolescent girls and young women in a cash transfer programme for schooling in Malawi**. *BMC Public Health* 2018, **18**(1):403.

13. Baird S, Chirwa E, McIntosh C, Özler B: **What happens once the intervention ends? The medium-term impacts of a cash transfer programme in Malawi, 3ie Impact Evaluation Report 27**. New Delhi: International Initiative for Impact Evaluation (3ie); 2015.

14. Kohler HP, Thornton R: **Conditional Cash Transfers and HIV/AIDS Prevention: Unconditionally Promising?** *The World Bank economic review* 2012, **26**(2):165-190.

15. Björkman Nyqvist M, Corno L, De Walque D, Svensson J: **Using Lotteries to Incentivize Safer Sexual Behavior : Evidence from a Randomized Controlled Trial on HIV Prevention. Policy Research Working Paper No. 7215**. Washington, DC: World Bank Group; 2015.

16. de Walque D, Dow WH, Nathan R, Abdul R, Abilahi F, Gong E, Isdahl Z, Jamison J, Jullu B, Krishnan S *et al*: **Incentivising safe sex: a randomised trial of conditional cash transfers for HIV and sexually transmitted infection prevention in rural Tanzania**. *BMJ Open* 2012, **2**(1).

17. De Walque D, Dow WH, Nathan R: **Rewarding Safer Sex: Conditional Cash Transfers for HIV/STI Prevention. Policy Research Working Paper;No. 7099**. In*.* Washington, DC: World Bank Group; 2014.

18. Handa S, Peterman A, Huang C, Halpern C, Pettifor A, Thirumurthy H: **Impact of the Kenya Cash Transfer for Orphans and Vulnerable Children on early pregnancy and marriage of adolescent girls**. *Soc Sci Med* 2015, **141**:36-45.

19. Heise L, Lutz B, Ranganathan M, Watts C: **Cash transfers for HIV prevention: considering their potential**. *Journal of the International AIDS Society* 2013, **16**(1):18615.

20. Handa S, Palermo T, Rosenberg M, Pettifor A, Halpern CT, Thirumurthy H: **How does a national poverty programme influence sexual debut among Kenyan adolescents?** *Global public health* 2017, **12**(5):617-638.

21. MacPhail C, Khoza N, Selin A, Julien A, Twine R, Wagner RG, Gomez-Olive X, Kahn K, Wang J, Pettifor A: **Cash transfers for HIV prevention: what do young women spend it on? Mixed methods findings from HPTN 068**. *BMC Public Health* 2017, **18**(1):10.

22. Khoza N, Stadler J, MacPhail C, Chikandiwa A, Brahmbhatt H, Delany-Moretlwe S: **Cash transfer interventions for sexual health: meanings and experiences of adolescent males and females in inner-city Johannesburg**. *BMC Public Health* 2018, **18**(1):120.
