# Supplementary material for: Spillover HIV prevention effects of a cash transfer trial in East Zimbabwe: evidence from a cluster-randomised trial and general-population survey
Source: BMC Public Health. 2020 Oct 23;20:1599. doi: 10.1186/s12889-020-09667-5 (PMC7584095; doi:10.1186/s12889-020-09667-5)
Supplement: Supplementary file 2 — Additional file 2. Contains the following sections, referred to throughout the article: 1. Additional information on data and measures (p.2). 2. Characteristics of the analysed sample compared to those not included (p.5). 3. Socio-demographic characteristics and balance of Trial groups (p.6). 4. Additional information on methods and results from propensity score matching (p.8) [file 12889_2020_9667_MOESM2_ESM.docx]

**Additional file 2**

**Spillover HIV prevention effects of a cash transfer trial on child development in East Zimbabwe: Evidence from a cluster-randomised trial and general-population survey**

**Schaefer et al.**

**BMC Public Health**

**This supplementary material contains:**

[1. Additional information on data and measures 2](#_Toc45816567)

[2. Characteristics of the analysed sample compared to those not included 5](#_Toc45816568)

[3. Socio-demographic characteristics and balance of Trial groups 6](#_Toc45816569)

[4. Additional information on methods and results from propensity score matching 8](#_Toc45816570)

[5. References 10](#_Toc45816571)

## 1. Additional information on data and measures

The analyses presented in the main article use data from the Manicaland Trial (2010-11) and the Manicaland Cohort survey in 2009-11. The main information taken from the Manicaland Trial was the allocation into the treatment (unconditional or conditional cash transfer) and control groups. The analyses of outcomes of the CT intervention presented in the main article were based on data from the Manicaland Cohort survey, analysed by intervention group of the Manicaland Trial. Data on socio-demographic characteristics from the Cohort were used to describe baseline balance between the treatment and control groups. Table S2.1 describes data used for these measures. Data used for all outcome measures (as listed in Tables 3 in the main article) are described in Table S2.2. The survey questionnaire is also available online (http://www.manicalandhivproject.org/questionnaires.html).

The only analysis that used data collected during the baseline survey of the Manicaland Trial was the comparison of individuals that were linked to the Cohort and those not linked in order to evaluate whether there were systematic differences between these groups. This analysis (see next section in this supplementary material) used data on socio-demographic background characteristics that were gathered with the same measurements as during the Manicaland Cohort. The only exceptions were chronic illness (for each member of the household, it was asked whether the person has a chronic illness) and orphan status (for each child under the age of 18 years living in the household, it was asked whether the biological mother and father are alive).

| Table S2.1 Survey measurement of socio-economic characteristics from the Manicaland Cohort, 2009-2011. | |
| --- | --- |
| **Variable** | **Measurement and variable creation** |
| **Educational attainment** | **Measurement:** What is the highest grade of school you have completed?  **Note:** The question allowed for ‘none’, ‘primary, ‘secondary, and ‘higher’ responses. As there were very few individuals with no or with higher education, two categories of educational attainment were created: ‘no or primary education’ and ‘secondary or higher education’. |
| **Marital status** | **Measurement:**  1) Have you ever been married or in a long-term or cohabiting relationship. Relationships of 12 months or more should be treated as “long-term”.  2) Are you currently widowed, divorced or separated from your most recent spouse/partner?  **Note:** Based on questions 1) and 2), individuals were classified as never married, currently married, separated/divorced, or widowed. |
| **Socio-economic status (wealth index)** | **Measurement:**  1) What is the main source of drinking water for members of your household?  2) What kind of toilet facility does your household have?  3) Is this toilet facility used by members of your household alone, shared with neighbours, or is it communal?  4) Does your household have: Electricity? A refrigerator? A radio? A television?  5) Record the house type.  6) Record the type of floor of the main dwelling.  7) Does any member of your household own: A bicycle? A motorcycle? A car? A tractor?  **Note:** A wealth index variable was created to represent socio-economic status. This was based on characteristics of and items present in the household, so it does not directly measure individual wealth. The index was based on sellable assets determined in question 4) and 7) and non-sellable assets determined in questions 1), 2), 3), 5), and 6); this index ranged from zero to one and was divided into quintiles. |
|  | |

| Table S2.2 Survey measurement of primary and secondary outcomes from the Manicaland Cohort, 2009-2011. | |
| --- | --- |
| **Variable** | **Measurement and variable creation** |
| **Primary outcomes** | |
| **Sexual debut** | **Measurement:** How old were you when you had sex for the first time?  **Note:** There was no specific question on sexual debut but the question on age at first sex allowed for a “Not yet had sex” response. |
| **Sex in past 30 days** | **Measurement:** How many days is it since you last had sex?  **Note:** Individuals were classified as having had sex in the past 30 days if they responded with 30 or fewer days. |
| **Multiple partners** | **Measurement:** How many different sexual partners have you had in the last 12 months?  **Note:** Individuals were classified as having multiple partners when reporting more than one sexual partner. |
| **Condom use** | **Measurement:** Did you use condoms throughout the last time you have sex?  **Note:** Individuals were classified as having used a condom if they responded positively. |
| **Secondary outcomes** | |
| **Psychological distress** | **Measurement:** In the past week… 1) Were you having headaches? 2) Was your appetite poor? 3) Were you having problems sleeping? 4) Did you have nightmares or bad dreams? 5) Were you easily frightened? 6) Did your hands shake? 7) Did you feel tense, nervous or worried? 8) Were you having digestion (tummy) problems? 9) Did you have trouble thinking clearly? 10) Did you sometimes think deeply or think about many things? 11) Did you cry more than usual? 12) Did you sometimes see or hear things which others could not see or hear? 13) Did you feel more unhappy than usual? 14) Did you have trouble enjoying your daily activities? 15) Did you find it difficult to make decisions? 16) Was your daily work suffering? 17) Did you find yourself sometimes failing to concentrate? 18) Did you lose your temper or get annoyed over trivial matters? 19) Were you able to play a useful part in life? 20) Did you lose interest in things? 21) Did you feel a worthless person? 22) Has the thought of ending your life been on your mind? 23) Did you have uncomfortable feelings in your stomach? 24) Were you feeling tired all the time? 25) Did you feel able to cope with most of the problems in your life?  **Note:** Psychological distress on an index of 25 questions relating to mental health, which is described in detail elsewhere [1]. Individuals were classified as psychologically distressed if they reported at least seven items of the scale. |
| **School enrolment** | **Measurement:** Are you currently enrolled in school full-time?  **Note:** Individuals were classified as being enrolled in school if they responded positively. |
| **Alcohol use** | **Measurement:**  1) How many times have you visited a bar or beer hall in the last month?  2) When you drink alcohol, do you usually have more than 3 beers / spirits etc. in one night?  **Note:** Individuals were classified as drinking alcohol if they responded positively to either of question 1) or 2). |
| **Cigarette smoking** | **Measurement:** Do you smoke cigarettes?  **Note:** Individuals were classified as current cigarette smokers if they responded positively. |
| **Recreational drug use** | **Measurement:** Do you take any drugs for please?  **Note:** Individuals were classified as taking recreational drugs if they reported any type of drug use (ingesting, injecting, or smoking). |
|  | |

## 2. Characteristics of the analysed sample compared to those not included

Data from the Trial baseline survey was used to evaluate whether there were systematic differences in socio-economic characteristics between individuals aged 15-54 years eligible for the Trial linked to the Cohort and those not linked. Based on data from the baseline survey of the Trial, there were significant differences between individuals included in this analysis (i.e. those linked to the Cohort) and those not included (Table S2.3). Those included tended to be older, more likely to be female and enrolled in school, of lower socio-economic status, more likely to have a chronic illness, and less likely to be orphaned. However, similar trends of these differences were observed in the control and treatment group, suggesting that no selection bias was introduced at the stage of linking the Trial and Cohort data.

| Table S2.3 Socio-demographic characteristics of individuals included and not included in this analysis, Manicaland Cash Transfer Trial, Manicaland, Zimbabwe, 2010-2011. | | | | | | |
| --- | --- | --- | --- | --- | --- | --- |
|  | Control | | | Treatment | | |
|  | Not included | Included |  | Not included | Included |  |
|  | % (95% CI) | % (95% CI) | %bias | % (95% CI) | % (95% CI) | %bias |
| Age |  |  |  |  |  |  |
| <20 years | 30.5 (28.7-32.5) | 24.8 (22.1-27.8) | 12.8 | 32.8 (31.6-34.0) | 28.1 (26.2-30.1) | 10.2 |
| 20-24 years | 22.2 (20.5-23.9) | 13.0 (11.0-15.4) | 24.1 | 22.1 (21.0-23.2) | 11.8 (10.4-13.2) | 27.8 |
| 25-29 years | 16.6 (15.1-18.2) | 12.6 (10.5-15.0) | 11.3 | 14.2 (13.4-15.2) | 10.5 (9.23-11.9) | 11.5 |
| 30-34 years | 10.1 (8.95-11.5) | 10.3 (8.45-12.5) | 0.54 | 9.16 (8.44-9.94) | 11.0 (9.77-12.5) | 6.23 |
| 35-39 years | 7.79 (6.75-8.98) | 12.9 (10.9-15.3) | 16.9 | 7.03 (6.39-7.72) | 11.1 (9.86-12.6) | 14.3 |
| 40-44 years | 4.32 (3.55-5.25) | 7.44 (5.87-9.38) | 13.3 | 5.33 (4.78-5.95) | 9.56 (8.38-10.9) | 16.2 |
| 45-49 years | 4.01 (3.27-4.91) | 10.8 (8.86-13.0) | 26.0 | 4.97 (4.43-5.56) | 9.61 (8.42-10.9) | 17.9 |
| 50-54 years | 4.46 (3.67-5.40) | 8.12 (6.48-10.1) | 15.1 | 4.37 (3.87-4.93) | 8.33 (7.22-9.59) | 16.3 |
|  |  |  |  |  |  |  |
| Sex: Male | 48.6 (46.5-50.6) | 36.3 (33.1-39.5) | 25.0 | 47.7 (46.4-49) | 37.5 (35.5-39.6) | 20.7 |
|  |  |  |  |  |  |  |
| Enrolled in school | 22.8 (19.0-27.1) | 16.0 (11.1-22.4) | 17.3 | 26.1 (23.6-28.7) | 14.4 (11.3-18.0) | 29.5 |
|  |  |  |  |  |  |  |
| SES (wealth index) |  |  |  |  |  |  |
| Poorest quarter | 42.7 (40.6-44.8) | 50.8 (47.5-54.1) | 16.3 | 48.3 (47.0-49.6) | 52.7 (50.6-54.9) | 8.91 |
| 2^nd^ quarter | 56.7 (54.6-58.8) | 47.6 (44.3-50.9) | 18.3 | 51.3 (50.0-52.6) | 46.5 (44.4-48.7) | 9.54 |
| 3^rd^ quarter | 0.65 (0.38-1.09) | 1.61 (0.96-2.71) | 9.15 | 0.41 (0.27-0.61) | 0.72 (0.43-1.19) | 4.18 |
| Least poor quarter | 0 | 0 |  | 0 | 0 |  |
|  |  |  |  |  |  |  |
| Chronic illness | 11.1 (9.84-12.5) | 15.4 (13.1-18.0) | 12.7 | 11.5 (10.7-12.4) | 17.7 (16.2-19.4) | 17.7 |
|  |  |  |  |  |  |  |
| Orphanhood ^[a]^ |  |  |  |  |  |  |
| Both parents alive | 30.8 (26.5-35.5) | 38.4 (31.3-45.9) | 15.9 | 32.7 (30.0-35.4) | 36.2 (31.8-40.9) | 7.53 |
| Father dead | 36.7 (32.1-41.5) | 33.1 (26.5-40.6) | 7.41 | 34.2 (31.5-36.9) | 36.9 (32.5-41.7) | 5.76 |
| Mother dead | 7.09 (4.96-10.0) | 6.98 (3.98-11.9) | 0.44 | 6.62 (5.33-8.19) | 5.18 (3.43-7.75) | 6.11 |
| Both parents dead | 25.4 (21.4-29.9) | 21.5 (16.0-28.4) | 9.23 | 26.5 (24.1-29.1) | 21.6 (18.0-25.8) | 11.5 |
| CI: Confidence interval; %bias: Standardised percent bias  Numbers are percentages (%) and 95% CI for different characteristics of those included in the analysis and those not included, separately for the control and treatment (cash transfer) group. The comparison is based on the baseline household survey of the Manicaland Trial in which one individual (the head of household) provided data on everyone living in the household. Those included in this analysis are those individuals linked to the Manicaland Cohort. The comparison excluded those aged under 15 years and over 54 years and those not eligible for the Trial.  ^[a]^ Restricted to those aged under 18 years. | | | | | | |

## 3. Socio-demographic characteristics and balance of Trial groups

To evaluate cluster balance, preliminary analyses described Trial groups in terms of socio-economic characteristics that were unlikely to be affected by the intervention or change within short time periods (age, sex, education, marital status, and socio-economic status) and HIV prevalence (which was not expected to be affected within the short period of the Trial). Standardised percent biases were calculated. [2] For both age groups, treatment and control groups were balanced across socio-demographic characteristics except for age and socio-economic status, with, on average, slightly higher socio-economic status in the control group (Table S2.4). Therefore, all regressions adjusted for age and socio-economic status (determined in the Manicaland Cohort). The same covariates were included in analyses with the synthetic comparison group for consistency, although good balance across all characteristics was achieved between synthetic comparison and original treatment groups (see next section).

| Table S2.4 Socio-demographic characteristics and HIV prevalence of Trial groups, Manicaland Cash Transfer Trial, Manicaland, Zimbabwe, 2010-2011. | | | | | | |
| --- | --- | --- | --- | --- | --- | --- |
|  | **15-29 years** | | | **30-54 years** | | |
|  | **Control** | **Treatment** |  | **Control** | **Treatment** |  |
|  | **% (95% CI)** | **% (95% CI)** | **%bias** | **% (95% CI)** | **% (95% CI)** | **%bias** |
| Age |  |  |  |  |  |  |
| <20 years | 58.4 (53.8-62.9) | 61.2 (58.3-64.0) | 5.63 |  |  |  |
| 20-24 years | 20.6 (17.1-24.5) | 22.1 (19.8-24.6) | 3.74 |  |  |  |
| 25-29 years | 21.0 (17.5-25.0) | 16.7 (14.6-19.0) | 11.0 |  |  |  |
| 30-34 years |  |  |  | 19.2 (15.6-23.4) | 19.0 (16.6-21.6) | 0.71 |
| 35-39 years |  |  |  | 26.1 (22.0-30.7) | 23.1 (20.5-25.9) | 6.92 |
| 40-44 years |  |  |  | 19.2 (15.6-23.4) | 19.0 (16.6-21.6) | 0.71 |
| 45-49 years |  |  |  | 16.7 (13.3-20.7) | 20.3 (17.9-23.0) | 9.35 |
| 50-54 years |  |  |  | 18.7 (15.2-22.9) | 18.6 (16.3-21.3) | 0.23 |
|  |  |  |  |  |  |  |
| Sex: Male | 50.8 (46.2-55.3) | 48.1 (45.1-51.0) | 5.39 | 23.8 (19.8-28.3) | 25.7 (23-28.6) | 4.50 |
|  |  |  |  |  |  |  |
| Education: |  |  |  |  |  |  |
| None/primary | 14.3 (11.3-17.8) | 10.8 (9.09-12.7) | 10.5 | 48.4 (43.4-53.5) | 48.7 (45.5-52) | 0.58 |
|  |  |  |  |  |  |  |
| Socio-economic status (wealth index)^a^ |  |  |  |  |  |  |
| Poorest quarter | 31.3 (27.2-35.7) | 37.3 (34.5-40.2) | 12.6 | 35.2 (30.6-40.1) | 40.9 (37.8-44.1) | 11.8 |
| 2^nd^ quarter | 62.1 (57.6-66.5) | 57.1 (54.1-59.9) | 10.4 | 57.2 (52.3-62.0) | 55.8 (52.6-59.0) | 2.80 |
| 3^rd^ quarter | 6.56 (4.62-9.25) | 5.21 (4.05-6.69) | 5.75 | 7.59 (5.35-10.7) | 3.07 (2.14-4.39) | 20.2 |
| Least poor quarter | 0 | 0.45 (0.19-1.08) |  | 0 | 0.21 (0.05-0.85) |  |
|  |  |  |  |  |  |  |
| Marital status |  |  |  |  |  |  |
| Never married | 70.0 (65.6-74.0) | 71.0 (68.2-73.6) | 2.18 | 2.53 (1.36-4.65) | 2.33 (1.54-3.52) | 1.29 |
| Currently married | 25.4 (21.6-29.7) | 23.9 (21.5-26.5) | 3.52 | 62 (57.1-66.7) | 61.4 (58.2-64.5) | 1.29 |
| Separated/divorced | 2.41 (1.34-4.31) | 3.51 (2.57-4.77) | 6.46 | 10.1 (7.51-13.5) | 7.1 (5.63-8.93) | 10.8 |
| Widowed | 2.19 (1.18-4.04) | 1.62 (1.02-2.56) | 4.20 | 25.3 (21.3-29.9) | 29.2 (26.3-32.2) | 8.64 |
|  |  |  |  |  |  |  |
| HIV positive | 7.02 (5.00-9.77) | 6.70 (5.37-8.34) | 1.24 | 32.7 (28.3-37.6) | 32.8 (29.9-35.9) | 0.12 |
| CI: Confidence interval; % bias: Standardised percent bias  Numbers are percentages (%) and 95% CI for different characteristics of the control and treatment (cash transfer) groups of the Manicaland Trial included in this analysis and the standardised percent bias of the groups, separately by age group. Data on all variables were taken from the Manicaland Cohort survey.  ^a^ Socio-economic status was evaluated with a wealth index that was based on a range of household assets. The index could take on values between 0 and 1 and individuals were divided into quarters. | | | | | | |

## 4. Additional information on methods and results from propensity score matching

The main article presented analyses of a synthetic comparison group that was created from Manicaland Cohort participants that did not live in Trial households through propensity score matching (PSM) [3]. In this section, more details on and results from PSM are presented.

Propensity scores (PS) were estimated using logistic regressions including a range of background characteristics to optimally balance the original treatment and synthetic comparison group. In preliminary analyses, different background characteristics were considered (age, marital status, educational attainment, socio-economic status, HIV status) (using Manicaland Cohort data). PS were estimated with different combinations of these variables and the balance of the synthetic comparison group and original treatment group were examined with descriptive statistics and standardised percentage bias (as proposed by Austin [4]). Using statistical tests to evaluate balance of matched samples is not recommended (see [5-7] for discussion). The set of variables in which the median standardised percentage bias across variables was lowest included age, marital status, socio-economic status, and HIV status.

To achieve perfect balance of the socio-economically different study sites, a ‘within site’ approach was taken [8-10]: Separately for each study site, PS were estimated and Trial treatment individuals were matched to Cohort individuals. PSM was implemented with nearest-neighbour matching with replacement, i.e. allowing the same Cohort individual being matched to several Trial individuals (which has been found to reduce bias compared to 1:1 matching [11]), imposing a calliper of 0.2 of the standard deviation of the PS (as recommended by Austin [12]). Ties were allowed, i.e. several Cohort individuals with the same PS could be matched to the same Trial individual, because the PS estimation included several categorical variables and equal PS for several individuals were expected.

PSM was implemented by sex and the same age groups as for the analyses of the original Trial sample. After establishing the synthetic comparison group, the same analyses were performed as for the original Trial data, i.e. ATEs were estimated based on mixed-effects logistic regressions (for a discussion of this approach see [7, 13, 14]), by sex and age group. This approach accounts for the clustering in the data, although standard errors in regressions were inaccurate because they did not account for the estimation of the PS [4, 15]; however, standard errors in this approach are typically conservative [16] (and proposed standard error corrections to account for the PS estimation [17] do not account for the clustering in the data).

There were also limitations in methods used in this study to estimate results with the synthetic comparison group that did not take into account the fact PS in the PSM were estimated [4, 15], thus underestimating standard errors. However, the approach to take into account that responses within clusters and study sites are correlated tends to result in conservative standard errors [16]. Moreover, standard errors may be underestimated in this study because individual-level, not cluster-level, data were used. While the strength of effects on key outcomes of this study and consistency with which these were found provides some confidence that these represent real effects, caution should be applied to effect sizes with large uncertainty.

Good balance across all socio-demographic characteristics was achieved between the synthetic comparison and original treatment groups for younger and older individuals after PSM (Table S2.5). However, the same covariates as were used for analyses of the original Trial data (age and socio-economic status) were included in analyses with the synthetic comparison group for consistency.

| Table S2.5 Balance of synthetic comparison group and original Trial treatment group, Manicaland Cash Transfer Trial, Manicaland, Zimbabwe, 2010-2011. | | | | | | |
| --- | --- | --- | --- | --- | --- | --- |
|  | 15-29 years | | | 30-54 years | | |
|  | Synthetic control | Treatment |  | Synthetic control | Treatment |  |
|  | % (95% CI) | % (95% CI) | %bias | % (95% CI) | % (95% CI) | %bias |
| Age |  |  |  |  |  |  |
| <20 years | 63.5 (60.3-66.6) | 61.4 (58.5-64.2) | 4.31 |  |  |  |
| 20-24 years | 22.8 (20.2-25.7) | 22.1 (19.8-24.7) | 1.75 |  |  |  |
| 25-29 years | 13.7 (11.5-16.2) | 16.5 (14.4-18.8) | 7.77 |  |  |  |
| 30-34 years |  |  |  | 19.8 (16.7-23.3) | 18.6 (16.2-21.2) | 2.83 |
| 35-39 years |  |  |  | 23.6 (20.0-27.7) | 23.1 (20.5-25.9) | 1.26 |
| 40-44 years |  |  |  | 19.1 (15.9-22.9) | 19.1 (16.7-21.8) | 0.00 |
| 45-49 years |  |  |  | 17.2 (13.7-21.5) | 20.5 (18.1-23.3) | 8.78 |
| 50-54 years |  |  |  | 20.2 (15.8-25.5) | 18.6 (16.2-21.2) | 4.26 |
|  |  |  |  |  |  |  |
| Sex: Male | 49.8 (46.5-53.0) | 47.7 (44.7-50.6) | 4.18 | 24.5 (20.9-28.5) | 25.2 (22.6-28.1) | 1.71 |
|  |  |  |  |  |  |  |
| Education: |  |  |  |  |  |  |
| None/primary | 12.7 (10.4-15.4) | 10.8 (9.09-12.8) | 6.38 | 51.4 (46.5-56.2) | 48.9 (45.6-52.2) | 4.97 |
|  |  |  |  |  |  |  |
| SES (wealth index) |  |  |  |  |  |  |
| Poorest quarter | 35.7 (32.4-39.2) | 37.2 (34.4-40.1) | 3.21 | 41.0 (36.2-45.9) | 41.0 (37.8-44.2) | 0.00 |
| 2^nd^ quarter | 59.0 (55.6-62.3) | 57.1 (54.2-60.0) | 4.02 | 55.7 (50.8-60.5) | 55.7 (52.5-58.9) | 0.00 |
| 3^rd^ quarter | 5.17 (4.08-6.52) | 5.26 (4.08-6.75) | 0.40 | 3.21 (2.05-4.99) | 3.10 (2.16-4.43) | 0.64 |
| Least poor quarter | 0.09 (0.01-0.64) | 0.45 (0.19-1.09) | 7.21 | 0.11 (0.01-0.76) | 0.21 (0.05-0.85) | 2.67 |
|  |  |  |  |  |  |  |
| Marital status |  |  |  |  |  |  |
| Never married | 73.2 (70.3-75.8) | 71.0 (68.2-73.6) | 4.80 | 1.39 (0.75-2.56) | 2.35 (1.55-3.55) | 7.21 |
| Currently married | 23.3 (20.9-25.9) | 23.8 (21.4-26.5) | 1.25 | 60.9 (55.6-65.9) | 61.1 (57.9-64.2) | 0.47 |
| Separated/divorced | 2.45 (1.50-3.97) | 3.54 (2.59-4.81) | 7.22 | 8.24 (5.69-11.8) | 7.17 (5.68-9.01) | 4.44 |
| Widowed | 1.09 (0.43-2.70) | 1.63 (1.03-2.58) | 5.53 | 29.5 (24.6-35.0) | 29.4 (26.6-32.4) | 0.26 |
|  |  |  |  |  |  |  |
| HIV positive | 5.53 (3.77-8.04) | 6.71 (5.37-8.35) | 5.67 | 30.7 (25.8-36) | 32.8 (29.9-35.9) | 4.98 |
| CI: Confidence interval; %bias: Standardised percent bias  Numbers are percentages (%) and 95% CI for different characteristics of the synthetic control and treatment (cash transfer) groups of the Manicaland Trial included in this analysis and the standardised percent bias of the groups, separately by age group. Data on all variables were taken from the Manicaland Cohort survey. The control group was determined through propensity score matching of individuals from the Manicaland Cohort to treatment-group individuals from the Manicaland Trial. | | | | | | |

## 5. References

1. Tlhajoane M, Eaton JW, Takaruza A, Rhead R, Maswera R, Schur N, Sherr L, Nyamukapa C, Gregson S: **Prevalence and Associations of Psychological Distress, HIV Infection and HIV Care Service Utilization in East Zimbabwe**. *AIDS and Behavior* 2018, **22**(5):1485-1495.

2. Rosenbaum PR, Rubin DB: **Constructing a Control Group Using Multivariate Matched Sampling Methods That Incorporate the Propensity Score**. *The American Statistician* 1985, **39**(1):33-38.

3. Rosenbaum PR, Rubin DB: **The central role of the propensity score in observational studies for causal effects**. *Biometrika* 1983, **70**(1):41-55.

4. Austin PC: **Balance diagnostics for comparing the distribution of baseline covariates between treatment groups in propensity-score matched samples**. *Statistics in medicine* 2009, **28**(25):3083-3107.

5. Imai K, King G, Stuart EA: **Misunderstandings between experimentalists and observationalists about causal inference**. *Journal of the Royal Statistical Society: Series A (Statistics in Society)* 2008, **171**(2):481-502.

6. Austin PC: **An Introduction to Propensity Score Methods for Reducing the Effects of Confounding in Observational Studies**. *Multivariate behavioral research* 2011, **46**(3):399-424.

7. Ho DE, Imai K, King G, Stuart EA: **Matching as Nonparametric Preprocessing for Reducing Model Dependence in Parametric Causal Inference**. *Political Analysis* 2007, **15**(3):199-236.

8. Steiner PM, Kim J-S, Thoemmes FJ: **Matching Strategies for Observational Multilevel Data** In: *JSM Proceedings.* edn. Alexandria, VA: American Statistical Association; 2012: 5020-5032.

9. Arpino B, Cannas M: **Propensity score matching with clustered data. An application to the estimation of the impact of caesarean section on the Apgar score**. *Statistics in medicine* 2016, **35**(12):2074-2091.

10. Arpino B, Mealli F: **The specification of the propensity score in multilevel observational studies**. *Computational Statistics & Data Analysis* 2011, **55**(4):1770-1780.

11. Ming K, Rosenbaum PR: **Substantial Gains in Bias Reduction from Matching with a Variable Number of Controls**. *Biometrics* 2000, **56**(1):118-124.

12. Austin PC: **Optimal caliper widths for propensity-score matching when estimating differences in means and differences in proportions in observational studies**. *Pharmaceutical Statistics* 2011, **10**(2):150-161.

13. DuGoff EH, Schuler M, Stuart EA: **Generalizing Observational Study Results: Applying Propensity Score Methods to Complex Surveys**. *Health Services Research* 2014, **49**(1):284-303.

14. Thoemmes FJ, West SG: **The Use of Propensity Scores for Nonrandomized Designs With Clustered Data**. *Multivariate Behavioral Research* 2011, **46**(3):514-543.

15. Caliendo M, Kopeinig S: **SOME PRACTICAL GUIDANCE FOR THE IMPLEMENTATION OF PROPENSITY SCORE MATCHING**. *Journal of Economic Surveys* 2008, **22**(1):31-72.

16. Wooldridge JM: **Inverse probability weighted estimation for general missing data problems**. *Journal of Econometrics* 2007, **141**(2):1281-1301.

17. Abadie A, Imbens GW: **Large Sample Properties of Matching Estimators for Average Treatment Effects**. *Econometrica* 2006, **74**(1):235-267.
