# Supplementary material for: Spillover HIV prevention effects of a cash transfer trial in East Zimbabwe: evidence from a cluster-randomised trial and general-population survey
Source: BMC Public Health. 2020 Oct 23;20:1599. doi: 10.1186/s12889-020-09667-5 (PMC7584095; doi:10.1186/s12889-020-09667-5)
Supplement: Supplementary file 3 — Additional file 3 Contains the following sections, referred to throughout the article: 1. Sample sizes and p-values for main results tables (p.2). 2. Results by type of CT intervention (CCT and UCT) (p.7). 3. Additional results by type of sex of head of household (p.12) [file 12889_2020_9667_MOESM3_ESM.docx]

**Additional file 3**

**Spillover HIV prevention effects of a cash transfer trial on child development in East Zimbabwe: Evidence from a cluster-randomised trial and general-population survey**

**Schaefer et al.**

**BMC Public Health**

**This supplementary material contains:**

[1. Sample sizes and p-values for main results tables 2](#_Toc45816651)

[2. Results by type of CT intervention (CCT and UCT) 7](#_Toc45816652)

[3. Additional results by type of sex of head of household 12](#_Toc45816653)

## 1. Sample sizes and p-values for main results tables

The results tables in the main article do not provide p-values for regressions or sample sizes for comparisons with synthetic comparison groups. In this section, the full details on the results are provided, with the tables showing the following:

- Table S3.1: Full results for young people using the original Trial data.
- Table S3.2: Full results for young people when compared to the synthetic comparison group.
- Table S3.3: Full results for older people using the original Trial data.
- Table S3.4: Full results for older people when compared to the synthetic comparison group.

| Table S3.1 Effects of cash transfers on young people (15-29 years), against the original comparison group, Manicaland Cash Transfer Trial, Manicaland, Zimbabwe, 2010-2011. | | | | | | | | | | |
| --- | --- | --- | --- | --- | --- | --- | --- | --- | --- | --- |
|  | Males (15-29) | | | | | Females (15-29) | | | | |
|  | Ctrl | CT | CT vs. ctrl (reference) | | | Ctrl | CT | CT vs. ctrl (reference) | | |
| Outcome: | n/N (%) | n/N (%) | ATE | (95% CI) | p-value | n/N (%) | n/N (%) | ATE | (95% CI) | p-value |
| **Primary outcomes** | | | | | | | | | | |
| Had sexual debut ^[a]^ | 7/156 (4.49) | 28/391 (7.16) | 1.43 | (-3.18, 6.05) | 0.543 | 20/126 (15.9) | 65/349 (18.6) | 2.63 | (-4.11, 9.38) | 0.444 |
| Had sex in past 30 days | 41/57 (71.9) | 69/122 (56.6) | -11.7 | (-26.0, 2.61) | 0.109 | 82/110 (74.6) | 197/282 (69.9) | -5.68 | (-15.7, 4.35) | 0.267 |
| Condom use (last sex) | 21/57 (36.8) | 51/122 (41.8) | 2.68 | (-11.6, 17.0) | 0.713 | 19/110 (17.3) | 46/282 (16.3) | -0.19 | (-8.24, 7.86) | 0.963 |
| Multiple partners (past 12 months) | 10/57 (17.5) | 31/122 (25.4) | 8.49 | (-5.40, 22.4) | 0.231 | 0/110 (0.00) | 7/281 (2.49) | NA ^[b]^ |  |  |
| Transactional sex ^[c]^ | 3/57 (5.26) | 4/121 (3.31) | -0.79 | (-6.31, 4.73) | 0.780 | 0/110 (0.00) | 4/282 (1.42) | NA ^[b]^ |  |  |
| Has a partner 5+ years older |  |  |  |  |  | 65/109 (59.6) | 164/264 (62.1) | 2.58 | (-8.34, 13.5) | 0.644 |
| **Secondary outcomes** | | | | | | | | | | |
| Psychological distress ^[d]^ | 29/232 (12.5) | 58/535 (10.8) | -1.20 | (-8.09, 5.69) | 0.733 | 52/225 (23.1) | 116/578 (20.1) | -2.11 | (-8.68, 4.46) | 0.529 |
| Self-efficacy ^[e]^ | 188/232 (81.0) | 436/535 (81.5) | 0.67 | (-8.01, 9.35) | 0.880 | 176/225 (78.2) | 481/578 (83.2) | 2.43 | (-6.09, 11.0) | 0.576 |
| Risk perception ^[f]^ | 28/230 (12.2) | 48/534 (8.99) | -1.43 | (-7.82, 4.95) | 0.660 | 47/220 (21.4) | 131/558 (23.5) | 3.02 | (-3.34, 9.39) | 0.352 |
| Had HIV test in past 12 months | 41/232 (17.7) | 66/534 (12.4) | -3.10 | (-9.26, 3.06) | 0.324 | 98/225 (43.6) | 221/577 (38.3) | -2.58 | (-9.58, 4.41) | 0.469 |
| Is currently enrolled in school ^[a]^ | 101/156 (64.7) | 281/391 (71.9) | 11.5 | (3.05, 19.9) | 0.008 | 82/126 (65.1) | 227/349 (65.0) | -1.21 | (-10.5, 8.07) | 0.799 |
| Alcohol use ^[g]^ | 34/232 (14.7) | 64/535 (12.0) | -0.03 | (-4.49, 4.43) | 0.991 | 0/225 (0.00) | 2/578 (0.35) | NA ^[b]^ |  |  |
| Smokes cigarettes | 20/232 (8.62) | 36/534 (6.74) | -0.38 | (-4.06, 3.30) | 0.841 | 1/225 (0.44) | 0/578 (0.00) | NA ^[b]^ |  |  |
| Takes recreational drugs | 26/232 (11.2) | 47/533 (8.82) | -0.84 | (-6.47, 4.78) | 0.769 | 1/225 (0.44) | 1/578 (0.17) | NA ^[b]^ |  |  |
| Ctrl: Control; CT: Cash transfer; ATE: Average treatment effect; CI: Confidence interval  Sample: Young people (15-29 years) who had sex before for primary outcomes and all young people for secondary outcomes.  Numbers are sample sizes of individuals reporting the outcome among everyone with data on the outcome (n/N) together with percentages (%) in the control and treatment (CT) groups of the Manicaland Trial and the estimated ATEs with 95% CIs and p-values. ATEs for each outcome were estimated from separate mixed-effects logistic regression model, controlling for age and wealth index quarters (not shown), with study site and treatment cluster random effects.  ^[a]^ Analyses were restricted to those aged 15-20 years as it was not applicable to older individuals.  ^[b]^ No regression model was estimated due to sample size limitations.  ^[c]^ For males: Given any financial or material reward for sex in the past 12 months. For females: Received any financial or material reward for sex in the past 12 months.  ^[d]^ Reporting at least 7 symptoms of psychological distress of a 25-item scale.  ^[e]^ Reporting that there are things that can be done to prevent HIV infection.  ^[f]^ Perceiving a risk for HIV infection in the future.  ^[g]^ Having been to a beer hall (bar) in the past month or drinking more than 3 drinks when drinking alcohol. | | | | | | | | | | |

| Table S3.2 Effects of cash transfers on young people (15-29 years), against the synthetic comparison group, Manicaland Cash Transfer Trial, Manicaland, Zimbabwe, 2010-2011. | | | | | | | | | | |
| --- | --- | --- | --- | --- | --- | --- | --- | --- | --- | --- |
|  | Males (15-29) | | | | | Females (15-29) | | | | |
|  | S. ctrl | CT | CT vs. s. ctrl (reference) | | | S. ctrl | CT | CT vs. s. ctrl (reference) | | |
| Outcome: | n/N (%) | n/N (%) | ATE | (95% CI) | p-value | n/N (%) | n/N (%) | ATE | (95% CI) | p-value |
| **Primary outcomes** | | | | | | | | | | |
| Had sexual debut ^[a]^ | 40/390 (10.4) | 28/386 (7.25) | -1.62 | (-5.11, 1.87) | 0.362 | 61/366 (16.7) | 65/349 (18.6) | 2.92 | (-2.22, 8.05) | 0.266 |
| Had sex in past 30 days | 77/115 (67.5) | 65/115 (57.9) | -9.68 | (-13.1, -6.30) | <0.001 | 219/281 (77.7) | 196/281 (69.0) | -8.77 | (-16.3, -1.23) | 0.023 |
| Condom use (last sex) | 51/115 (43.8) | 49/115 (41.3) | -2.49 | (-9.70, 4.71) | 0.498 | 19/281 (7.04) | 45/281 (16.4) | 9.38 | (5.90, 12.9) | <0.001 |
| Multiple partners (past 12 months) | 17/115 (15.1) | 29/115 (25.3) | 10.3 | (1.27, 19.2) | 0.025 | 0/280 (0.08) | 7/280 (2.37) | NA ^[b]^ |  |  |
| Transactional sex ^[c]^ | 3/114 (1.77) | 4/114 (2.92) | 1.15 | (-3.72, 6.01) | 0.644 | 3/281 (0.58) | 4/281 (0.81) | NA ^[b]^ |  |  |
| Has a partner 5+ years older |  |  |  |  |  | 151/263 (57.0) | 163/263 (62.4) | 5.38 | (0.04, 10.7) | 0.048 |
| **Secondary outcomes** | | | | | | | | | | |
| Psychological distress ^[d]^ | 75/522 (13.6) | 57/522 (10.6) | -3.00 | (-6.75, 0.74) | 0.116 | 108/577 (19.3) | 116/577 (19.8) | 0.55 | (-4.64, 5.74) | 0.835 |
| Is currently enrolled in school ^[a]^ | 229/390 (61.2) | 277/386 (70.4) | 9.27 | (3.05, 15.5) | 0.003 | 214/366 (58.7) | 227/349 (64.2) | 5.50 | (1.62, 9.37) | 0.005 |
| Alcohol use ^[g]^ | 82/522 (15.7) | 61/522 (11.6) | -4.03 | (-8.49, 0.44) | 0.077 | 4/577 (0.70) | 2/577 (0.34) | NA ^[b]^ |  |  |
| Smokes cigarettes | 45/521 (8.24) | 35/521 (6.33) | -1.91 | (-5.03, 1.21) | 0.230 | 0/577 (0.00) | 0/577 (0.00) | NA ^[b]^ |  |  |
| Takes recreational drugs | 55/520 (10.1) | 45/520 (7.14) | -2.91 | (-6.78, 0.96) | 0.141 | 1/577 (0.34) | 1/577 (0.31) | NA ^[b]^ |  |  |
| S. ctrl: Synthetic control; CT: Cash transfer; ATE: Average treatment effect; CI: Confidence interval  Sample: Young people (15-29 years) who had sex before for primary outcomes and all young people for secondary outcomes. The synthetic comparison group was determined through propensity score matching of individuals from the Manicaland Cohort to treatment-group individuals from the Manicaland Trial.  Numbers are sample sizes of individuals reporting the outcome among everyone with data on the outcome (n/N) together with percentages (%) in the synthetic control and treatment (CT) groups of the Manicaland Trial and the estimated ATEs with 95% CIs and p-values. ATEs for each for each outcome were estimated from separate mixed-effects logistic regression model after propensity score matching, controlling for age and wealth index quarters (not shown), with study site and treatment cluster random effects. Propensity score matching for the control group was implemented with replacement. Probability weights were applied, so individuals may be counted several times, and numbers were rounded.  ^[a]^ Analyses were restricted to those aged 15-20 years as it was not applicable to older individuals.  ^[b]^ No regression model was estimated due to sample size limitations.  ^[c]^ For males: Given any financial or material reward for sex in the past 12 months. For females: Received any financial or material reward for sex in the past 12 months.  ^[d]^ Reporting at least 7 symptoms of psychological distress of a 25-item scale.  ^[e]^ Reporting that there are things that can be done to prevent HIV infection.  ^[f]^ Perceiving a risk for HIV infection in the future.  ^[g]^ Having been to a beer hall (bar) in the past month or drinking more than 3 drinks when drinking alcohol. | | | | | | | | | | |

| Table S3.3 Effects of cash transfers on older people (30-54 years), against the original comparison group, Manicaland Cash Transfer Trial, Manicaland, Zimbabwe, 2010-2011. | | | | | | | | | | |
| --- | --- | --- | --- | --- | --- | --- | --- | --- | --- | --- |
|  | Males (30-54) | | | | | Females (30-54) | | | | |
|  | Ctrl | CT | CT vs. ctrl (reference) | | | Ctrl | CT | CT vs. ctrl (reference) | | |
| Outcome: | n/N (%) | n/N (%) | ATE | (95% CI) | p-value | n/N (%) | n/N (%) | ATE | (95% CI) | p-value |
| **Primary outcomes** | | | | | | | | | | |
| Had sex in past 30 days | 84/92 (91.3) | 207/239 (86.6) | -5.05 | (-12.4, 2.25) | 0.175 | 152/300 (50.7) | 334/696 (48.0) | -1.61 | (-8.29, 5.07) | 0.636 |
| Condom use (last sex) | 22/92 (23.9) | 48/239 (20.1) | -2.30 | (-12.7, 8.08) | 0.664 | 67/300 (22.3) | 160/695 (23.0) | 1.16 | (-5.09, 7.42) | 0.715 |
| Multiple partners (past 12 months) | 13/92 (14.1) | 24/240 (10.0) | -2.84 | (-10.9, 5.26) | 0.492 | 5/300 (1.67) | 12/695 (1.73) | -0.40 | (-1.71, 0.91) | 0.554 |
| Transactional sex ^[a]^ | 3/90 (3.33) | 6/240 (2.50) | -0.06 | (-3.26, 3.15) | 0.973 | 6/299 (2.01) | 18/694 (2.59) | -0.16 | (-1.81, 1.50) | 0.853 |
| **Secondary outcomes** | | | | | | | | | | |
| Psychological distress ^[b]^ | 16/94 (17.0) | 41/243 (16.9) | -4.42 | (-15.7, 6.82) | 0.441 | 118/301 (39.2) | 254/701 (36.2) | -3.97 | (-11.7, 3.75) | 0.314 |
| Alcohol use ^[e]^ | 38/94 (40.4) | 104/243 (42.8) | 3.57 | (-11.2, 18.3) | 0.635 | 2/301 (0.66) | 9/700 (1.29) | NA ^[f]^ |  |  |
| Smokes cigarettes | 32/94 (34.0) | 80/243 (32.9) | -2.06 | (-14.8, 10.7) | 0.751 | 2/300 (0.67) | 5/700 (0.71) | NA ^[f]^ |  |  |
| Takes recreational drugs | 35/94 (37.2) | 63/242 (26.0) | -10.5 | (-23.5, 2.59) | 0.116 | 1/301 (0.33) | 8/697 (1.15) | NA ^[f]^ |  |  |
| Ctrl: Control; CT: Cash transfer; ATE: Average treatment effect; CI: Confidence interval  Sample: Older people (30-54 years) who had sex before for primary outcomes and all older people for secondary outcomes.  Numbers are sample sizes of individuals reporting the outcome among everyone with data on the outcome (n/N) together with percentages (%) in the control and treatment (CT) groups of the Manicaland Trial and the estimated ATEs with 95% CIs and p-values. ATEs for each outcome were estimated from separate mixed-effects logistic regression model, controlling for age and wealth index quarters (not shown), with study site and treatment cluster random effects.  ^[a]^ For males: Given any financial or material reward for sex in the past 12 months. For females: Received any financial or material reward for sex in the past 12 months.  ^[b]^ Reporting at least 7 symptoms of psychological distress of a 25-item scale.  ^[c]^ Reporting that there are things that can be done to prevent HIV infection.  ^[d]^ Perceiving a risk for HIV infection in the future.  ^[e]^ Having been to a beer hall (bar) in the past month or drinking more than 3 drinks when drinking alcohol.  ^[f]^ No regression model was estimated due to sample size limitations. | | | | | | | | | | |

| Table S3.4 Effects of cash transfers on older people (30-54 years), against the synthetic comparison group, Manicaland Cash Transfer Trial, Manicaland, Zimbabwe, 2010-2011. | | | | | | | | | | |
| --- | --- | --- | --- | --- | --- | --- | --- | --- | --- | --- |
|  | Males (15-29) | | | | | Females (15-29) | | | | |
|  | S. ctrl | CT | CT vs. s. ctrl (reference) | | | S. ctrl | CT | CT vs. s. ctrl (reference) | | |
| Outcome: | n/N (%) ^[a]^ | n/N (%) | ATE | (95% CI) | p-value | n/N (%) ^[a]^ | n/N (%) | ATE | (95% CI) | p-value |
| **Primary outcomes** | | | | | | | | | | |
| Had sex in past 30 days | 199/229 (87.5) | 198/229 (87.2) | -0.25 | (-8.56, 8.05) | 0.952 | 356/692 (51.7) | 332/692 (48.7) | -3.04 | (-10.3, 4.24) | 0.413 |
| Condom use (last sex) | 26/229 (11.4) | 44/229 (19.1) | 7.64 | (-0.57, 15.8) | 0.068 | 118/691 (16.5) | 158/691 (22.5) | 5.95 | (1.46, 10.4) | 0.009 |
| Multiple partners (past 12 months) | 28/230 (12.1) | 22/230 (9.52) | -2.54 | (-8.82, 3.73) | 0.427 | 9/691 (0.40) | 12/691 (0.42) | 0.02 | (-0.44, 0.47) | 0.946 |
| Transactional sex ^[a]^ | 10/230 (4.21) | 6/230 (2.68) | -1.54 | (-5.81, 2.74) | 0.481 | 6/690 (0.31) | 18/690 (0.93) | 0.63 | (-0.03, 1.29) | 0.062 |
| **Secondary outcomes** | | | | | | | | | | |
| Psychological distress ^[b]^ | 36/233 (14.2) | 39/233 (15.4) | 1.27 | (-5.33, 7.88) | 0.705 | 230/696 (33.0) | 253/696 (36.0) | 3.00 | (-1.62, 7.63) | 0.203 |
| Self-efficacy ^[c]^ | 203/233 (88.7) | 160/233 (70.9) | -17.8 | (-26.3, -9.35) | <0.001 | 582/696 (84.2) | 486/696 (70.3) | -13.9 | (-19.8, -8.00) | <0.001 |
| Risk perception ^[d]^ | 29/209 (13.2) | 15/209 (6.34) | -6.90 | (-13.4, -0.38) | 0.038 | 224/576 (38.6) | 220/576 (38.5) | -0.12 | (-4.96, 4.73) | 0.963 |
| Had HIV test in past 12 months | 56/232 (22.0) | 65/232 (26.3) | 4.37 | (-4.94, 13.7) | 0.358 | 304/696 (43.4) | 335/696 (48.2) | 4.88 | (-0.24, 9.99) | 0.062 |
| Alcohol use ^[e]^ | 120/233 (52.0) | 98/233 (41.3) | -10.7 | (-22.7, 1.22) | 0.078 | 24/695 (2.13) | 9/695 (0.72) | NA ^[f]^ |  |  |
| Smokes cigarettes | 85/233 (36.5) | 77/233 (32.5) | -4.05 | (-16.4, 8.27) | 0.520 | 0/695 (0.00) | 0/695 (0.00) | NA ^[f]^ |  |  |
| Takes recreational drugs | 67/232 (28.7) | 57/232 (23.9) | -4.80 | (-15.1, 5.53) | 0.363 | 7/692 (0.50) | 8/692 (0.66) | NA ^[f]^ |  |  |
| S. ctrl: Synthetic control; CT: Cash transfer; ATE: Average treatment effect; CI: Confidence interval  Sample: Older people (30-54 years) who had sex before for primary outcomes and all older people for secondary outcomes. The control group was determined through propensity score matching of individuals from the Manicaland Cohort to treatment-group individuals from the Manicaland Trial.  Numbers are sample sizes of individuals reporting the outcome among everyone with data on the outcome (n/N) together with percentages (%) in the synthetic control and treatment (CT) groups of the Manicaland Trial and the estimated ATEs with 95% CIs and p-values. ATEs for each outcome were estimated from separate mixed-effects logistic regression model after propensity score matching, controlling for age and wealth index quarters (not shown), with study site and treatment cluster random effects. Propensity score matching for the control group was implemented with replacement. Probability weights were applied, so individuals may be counted several times, and numbers were rounded.  ^[a]^ For males: Given any financial or material reward for sex in the past 12 months. For females: Received any financial or material reward for sex in the past 12 months.  ^[b]^ Reporting at least 7 symptoms of psychological distress of a 25-item scale.  ^[c]^ Reporting that there are things that can be done to prevent HIV infection.  ^[d]^ Perceiving a risk for HIV infection in the future.  ^[e]^ Having been to a beer hall (bar) in the past month or drinking more than 3 drinks when drinking alcohol.  ^[f]^ No regression model was estimated due to sample size limitations. | | | | | | | | | | |

## 2. Results by type of CT intervention (CCT and UCT)

In this section, results on the effects of unconditional and conditional cash transfers (UCTs and CCTs) among young people aged 15-29 years when compared to the original Trial comparison group and when compared to synthetic comparison groups are presented. The tables show the following:

- Table S3.5: Full results on effects by CCTs for young people using the original Trial data.
- Table S3.6: Full results on effects by UCTs for young people using the original Trial data.
- Table S3.7: Full results on effects by CCTs for young people when compared to the synthetic comparison group.
- Table S3.8: Full results on effects by UCTs for young people when compared to the synthetic comparison group.

| Table S3.5 Effects of conditional cash transfers on young people (15-29 years), Manicaland Cash Transfer Trial, Manicaland, Zimbabwe, 2010-2011. | | | | | | | | | | |
| --- | --- | --- | --- | --- | --- | --- | --- | --- | --- | --- |
|  | Males (15-29) | | | | | Females (15-29) | | | | |
|  | Ctrl | CCT | CCT vs. ctrl (reference) | | | Ctrl | CCT | CCT vs. ctrl (reference) | | |
| Outcome: | n/N (%) | n/N (%) | ATE | (95% CI) | p-value | n/N (%) | n/N (%) | ATE | (95% CI) | p-value |
| **Primary outcomes** | | | | | | | | | | |
| Had sexual debut ^[a]^ | 7/156 (4.49) | 16/199 (8.04) | 1.91 | (-3.31, 7.14) | 0.473 | 20/126 (15.9) | 34/193 (17.6) | 3.32 | (-4.15, 10.8) | 0.384 |
| Had sex in past 30 days | 41/57 (71.9) | 31/58 (53.5) | -15.3 | (-33.6, 3.04) | 0.102 | 82/110 (74.6) | 99/145 (68.3) | -6.63 | (-18.0, 4.77) | 0.254 |
| Condom use (last sex) | 21/57 (36.8) | 29/58 (50.0) | 8.42 | (-8.43, 25.3) | 0.327 | 19/110 (17.3) | 24/145 (16.6) | -0.03 | (-9.16, 9.09) | 0.994 |
| Multiple partners (past 12 months) | 10/57 (17.5) | 15/58 (25.9) | 7.10 | (-8.32, 22.5) | 0.367 | 0/110 (0.00) | 3/144 (2.08) | NA ^[b]^ |  |  |
| Transactional sex ^[c]^ | 3/57 (5.26) | 2/57 (3.51) | -1.37 | (-8.82, 6.08) | 0.719 | 0/110 (0.00) | 2/145 (1.38) | NA ^[b]^ |  |  |
| Has a partner 5+ years older |  |  |  |  |  | 65/109 (59.6) | 85/140 (60.7) | 0.27 | (-12.0, 12.5) | 0.966 |
| **Secondary outcomes** | | | | | | | | | | |
| Psychological distress ^[d]^ | 29/232 (12.5) | 27/266 (10.2) | -1.15 | (-9.34, 7.04) | 0.783 | 52/225 (23.1) | 52/311 (16.7) | -5.14 | (-11.8, 1.51) | 0.130 |
| Self-efficacy ^[e]^ | 188/232 (81.0) | 220/266 (82.7) | 1.66 | (-7.63, 11.0) | 0.726 | 176/225 (78.2) | 263/311 (84.6) | 3.01 | (-7.44, 13.5) | 0.572 |
| Risk perception ^[f]^ | 28/230 (12.2) | 28/266 (10.5) | -0.17 | (-7.99, 7.65) | 0.966 | 47/220 (21.4) | 73/302 (24.2) | 4.12 | (-2.92, 11.2) | 0.251 |
| Had HIV test in past 12 months | 41/232 (17.7) | 29/265 (10.9) | -4.16 | (-11.5, 3.17) | 0.266 | 98/225 (43.6) | 111/310 (35.8) | -4.88 | (-12.6, 2.84) | 0.215 |
| Is currently enrolled in school ^[a]^ | 101/156 (64.7) | 143/199 (71.9) | 13.0 | (5.19, 20.7) | 0.001 | 82/126 (65.1) | 129/193 (66.8) | -2.25 | (-12.9, 8.45) | 0.681 |
| Alcohol use ^[g]^ | 34/232 (14.7) | 29/266 (10.9) | -0.68 | (-6.01, 4.64) | 0.801 | 0/225 (0.00) | 1/311 (0.32) | NA ^[b]^ |  |  |
| Smokes cigarettes | 20/232 (8.62) | 16/266 (6.02) | -0.68 | (-5.15, 3.80) | 0.767 | 1/225 (0.44) | 0/311 (0.00) | NA ^[b]^ |  |  |
| Takes recreational drugs | 26/232 (11.2) | 24/265 (9.06) | -0.02 | (-5.57, 5.54) | 0.996 | 1/225 (0.44) | 1/311 (0.32) | NA ^[b]^ |  |  |
| Ctrl: Control; CCT: Conditional cash transfer; ATE: Average treatment effect; CI: Confidence interval  Sample: Young people (15-29 years) who had sex before for primary outcomes and all young people for secondary outcomes.  Numbers are sample sizes of individuals reporting the outcome among everyone with data on the outcome (n/N) together with percentages (%) in the control and treatment (CCT) groups of the Manicaland Trial and the estimated ATEs with 95% CIs and p-values. ATEs for each outcome were estimated from separate mixed-effects logistic regression model, controlling for age and wealth index quarters (not shown), with study site and treatment cluster random effects.  ^[a]^ Analyses were restricted to those aged 15-20 years as it was not applicable to older individuals.  ^[b]^ No regression model was estimated due to sample size limitations.  ^[c]^ For males: Given any financial or material reward for sex in the past 12 months. For females: Received any financial or material reward for sex in the past 12 months.  ^[d]^ Reporting at least 7 symptoms of psychological distress of a 25-item scale.  ^[e]^ Reporting that there are things that can be done to prevent HIV infection.  ^[f]^ Perceiving a risk for HIV infection in the future.  ^[g]^ Having been to a beer hall (bar) in the past month or drinking more than 3 drinks when drinking alcohol. | | | | | | | | | | |

| Table S3.6 Effects of unconditional cash transfers on young people (15-29 years), Manicaland Cash Transfer Trial, Manicaland, Zimbabwe, 2010-2011. | | | | | | | | | | |
| --- | --- | --- | --- | --- | --- | --- | --- | --- | --- | --- |
|  | Males (15-29) | | | | | Females (15-29) | | | | |
|  | Ctrl | UCT | UCT vs. ctrl (reference) | | | Ctrl | UCT | UCT vs. ctrl (reference) | | |
| Outcome: | n/N (%) | n/N (%) | ATE | (95% CI) | p-value | n/N (%) | n/N (%) | ATE | (95% CI) | p-value |
| **Primary outcomes** | | | | | | | | | | |
| Had sexual debut ^[a]^ | 7/156 (4.49) | 12/192 (6.25) | 0.67 | (-4.73, 6.06) | 0.809 | 20/126 (15.9) | 31/156 (19.9) | 2.15 | (-5.73, 10.0) | 0.593 |
| Had sex in past 30 days | 41/57 (71.9) | 38/64 (59.4) | -8.13 | (-24.6, 8.39) | 0.335 | 82/110 (74.6) | 98/137 (71.5) | -4.13 | (-15.8, 7.55) | 0.488 |
| Condom use (last sex) | 21/57 (36.8) | 22/64 (34.4) | -5.94 | (-22.6, 10.7) | 0.483 | 19/110 (17.3) | 22/137 (16.1) | -0.75 | (-9.67, 8.17) | 0.869 |
| Multiple partners (past 12 months) | 10/57 (17.5) | 16/64 (25.0) | 9.16 | (-5.32, 23.6) | 0.215 | 0/110 (0.00) | 4/137 (2.92) | NA ^[b]^ |  |  |
| Transactional sex ^[c]^ | 3/57 (5.26) | 2/64 (3.13) | -0.32 | (-4.08, 3.44) | 0.868 | 0/110 (0.00) | 2/137 (1.46) | NA ^[b]^ |  |  |
| Has a partner 5+ years older |  |  |  |  |  | 65/109 (59.6) | 79/124 (63.7) | 5.71 | (-7.05, 18.5) | 0.380 |
| **Secondary outcomes** | | | | | | | | | | |
| Psychological distress ^[d]^ | 29/232 (12.5) | 31/269 (11.5) | -1.13 | (-6.35, 4.09) | 0.671 | 52/225 (23.1) | 64/267 (24.0) | 1.49 | (-5.95, 8.93) | 0.695 |
| Self-efficacy ^[e]^ | 188/232 (81.0) | 216/269 (80.3) | 0.01 | (-10.7, 10.7) | 0.999 | 176/225 (78.2) | 218/267 (81.7) | -0.51 | (-11.9, 10.9) | 0.930 |
| Risk perception ^[f]^ | 28/230 (12.2) | 20/268 (7.46) | -2.64 | (-9.07, 3.8) | 0.422 | 47/220 (21.4) | 58/256 (22.7) | 1.46 | (-5.74, 8.66) | 0.691 |
| Had HIV test in past 12 months | 41/232 (17.7) | 37/269 (13.8) | -1.91 | (-8.8, 4.98) | 0.587 | 98/225 (43.6) | 110/267 (41.2) | -0.46 | (-8.65, 7.74) | 0.913 |
| Is currently enrolled in school ^[a]^ | 101/156 (64.7) | 138/192 (71.9) | 10.9 | (1.28, 20.5) | 0.026 | 82/126 (65.1) | 98/156 (62.8) | 0.13 | (-10.3, 10.5) | 0.980 |
| Alcohol use ^[g]^ | 34/232 (14.7) | 35/269 (13.0) | 0.27 | (-5.08, 5.61) | 0.922 | 0/225 (0.00) | 1/267 (0.37) | NA ^[b]^ |  |  |
| Smokes cigarettes | 20/232 (8.62) | 20/268 (7.46) | -0.40 | (-4.78, 3.98) | 0.858 | 1/225 (0.44) | 0/267 (0.00) | NA ^[b]^ |  |  |
| Takes recreational drugs | 26/232 (11.2) | 23/268 (8.58) | -2.15 | (-8.27, 3.96) | 0.490 | 1/225 (0.44) | 0/267 (0.00) | NA ^[b]^ |  |  |
| Ctrl: Control; UCT: Unconditional cash transfer; ATE: Average treatment effect; CI: Confidence interval  Sample: Young people (15-29 years) who had sex before for primary outcomes and all young people for secondary outcomes.  Numbers are sample sizes of individuals reporting the outcome among everyone with data on the outcome (n/N) together with percentages (%) in the control and treatment (UCT) groups of the Manicaland Trial and the estimated ATEs with 95% CIs and p-values. ATEs for each outcome were estimated from separate mixed-effects logistic regression model, controlling for age and wealth index quarters (not shown), with study site and treatment cluster random effects.  ^[a]^ Analyses were restricted to those aged 15-20 years as it was not applicable to older individuals.  ^[b]^ No regression model was estimated due to sample size limitations.  ^[c]^ For males: Given any financial or material reward for sex in the past 12 months. For females: Received any financial or material reward for sex in the past 12 months.  ^[d]^ Reporting at least 7 symptoms of psychological distress of a 25-item scale.  ^[e]^ Reporting that there are things that can be done to prevent HIV infection.  ^[f]^ Perceiving a risk for HIV infection in the future.  ^[g]^ Having been to a beer hall (bar) in the past month or drinking more than 3 drinks when drinking alcohol. | | | | | | | | | | |

| Table S3.7 Effects of conditional cash transfers on young people (15-29 years), against the synthetic comparison group, Manicaland Cash Transfer Trial, Manicaland, Zimbabwe, 2010-2011. | | | | | | | | | | |
| --- | --- | --- | --- | --- | --- | --- | --- | --- | --- | --- |
|  | Males (15-29) | | | | | Females (15-29) | | | | |
|  | S. ctrl | CCT | CCT vs. s. ctrl (reference) | | | S. ctrl | CCT | CCT vs. s. ctrl (reference) | | |
| Outcome: | n/N (%) | n/N (%) | ATE | (95% CI) | p-value | n/N (%) | n/N (%) | ATE | (95% CI) | p-value |
| **Primary outcomes** | | | | | | | | | | |
| Had sexual debut ^[a]^ | 18/204 (8.72) | 16/198 (8.08) | -0.40 | (-4.79, 3.99) | 0.858 | 33/199 (16.5) | 34/193 (17.6) | 4.13 | (-2.09, 10.3) | 0.193 |
| Had sex in past 30 days | 33/55 (58.2) | 29/55 (53.7) | -4.56 | (-18.3, 9.18) | 0.515 | 101/144 (70.1) | 98/144 (67.4) | -2.63 | (-12.3, 7.04) | 0.594 |
| Condom use (last sex) | 26/55 (49.1) | 28/55 (49.7) | 0.54 | (-8.61, 9.70) | 0.907 | 16/144 (10.9) | 23/144 (16.0) | 5.08 | (0.23, 9.93) | 0.040 |
| Multiple partners (past 12 months) | 12/55 (21.9) | 15/55 (27.1) | 5.17 | (-13.8, 24.2) | 0.594 | 0/143 (0.18) | 3/143 (2.26) | 2.08 | (0.14, 4.02) | 0.036 |
| Transactional sex ^[b]^ | 2/54 (3.74) | 2/54 (3.67) | -0.07 | (-5.76, 5.61) | 0.980 | 1/144 (0.59) | 2/144 (1.32) | 0.73 | (-0.88, 2.35) | 0.375 |
| Has a partner 5+ years older |  |  |  |  |  | 85/139 (60.5) | 84/139 (60.8) | 0.24 | (-12.6, 13.1) | 0.971 |
| **Secondary outcomes** | | | | | | | | | | |
| Psychological distress ^[c]^ | 39/261 (14.1) | 27/261 (9.88) | -4.21 | (-12.0, 3.62) | 0.292 | 62/310 (20.0) | 52/310 (16.7) | -3.36 | (-9.44, 2.71) | 0.277 |
| Self-efficacy ^[d]^ | 212/261 (81.4) | 215/261 (82.5) | 1.04 | (-3.73, 5.81) | 0.670 | 274/310 (88.2) | 263/310 (84.9) | -3.30 | (-7.54, 0.93) | 0.126 |
| Risk perception ^[e]^ | 28/261 (9.35) | 26/261 (7.22) | -2.14 | (-8.97, 4.69) | 0.540 | 79/301 (26.4) | 72/301 (24.1) | -2.30 | (-9.50, 4.90) | 0.531 |
| Had HIV test in past 12 months | 27/260 (9.80) | 29/260 (10.4) | 0.63 | (-4.70, 5.96) | 0.817 | 107/309 (34.6) | 111/309 (35.9) | 1.23 | (-3.06, 5.52) | 0.575 |
| Is currently enrolled in school ^[a]^ | 123/204 (61.1) | 142/198 (71.7) | 10.6 | (3.28, 17.9) | 0.004 | 115/199 (59.3) | 129/193 (64.0) | 4.72 | (-1.36, 10.8) | 0.128 |
| Alcohol use ^[f]^ | 38/261 (14.6) | 28/261 (10.1) | -4.53 | (-10.5, 1.45) | 0.138 | 1/310 (0.67) | 1/310 (0.46) | -0.20 | (-1.60, 1.19) | 0.773 |
| Smokes cigarettes | 21/261 (7.00) | 15/261 (4.51) | -2.49 | (-5.25, 0.26) | 0.076 | 0/310 (0.00) | 0/310 (0.00) | NA ^[g]^ |  |  |
| Takes recreational drugs | 24/260 (8.22) | 23/260 (7.02) | -1.20 | (-4.05, 1.65) | 0.410 | 0/310 (0.16) | 1/310 (0.55) | 0.39 | (-0.78, 1.56) | 0.512 |
| S. ctrl: Synthetic control; CCT: Conditional cash transfer; ATE: Average treatment effect; CI: Confidence interval  Sample: Young people (15-29 years) who had sex before for primary outcomes and all young people for secondary outcomes. The synthetic comparison group was determined through propensity score matching of individuals from the Manicaland Cohort to treatment-group individuals from the Manicaland Trial.  Numbers are sample sizes of individuals reporting the outcome among everyone with data on the outcome (n/N) together with percentages (%) in the synthetic control and treatment (CCT) groups of the Manicaland Trial and the estimated ATEs with 95% CIs and p-values. ATEs for each for each outcome were estimated from separate mixed-effects logistic regression model after propensity score matching, controlling for age and wealth index quarters (not shown), with study site and treatment cluster random effects. Propensity score matching for the control group was implemented with replacement. Probability weights were applied, so individuals may be counted several times, and numbers were rounded.  ^[a]^ Analyses were restricted to those aged 15-20 years as it was not applicable to older individuals.  ^[b]^ For males: Given any financial or material reward for sex in the past 12 months. For females: Received any financial or material reward for sex in the past 12 months.  ^[c]^ Reporting at least 7 symptoms of psychological distress of a 25-item scale.  ^[d]^ Reporting that there are things that can be done to prevent HIV infection.  ^[e]^ Perceiving a risk for HIV infection in the future.  ^[f]^ Having been to a beer hall (bar) in the past month or drinking more than 3 drinks when drinking alcohol.  ^[g]^ No regression model was estimated due to sample size limitations. | | | | | | | | | | |

| Table S3.8 Effects of unconditional cash transfers on young people (15-29 years), against the synthetic comparison group, Manicaland Cash Transfer Trial, Manicaland, Zimbabwe, 2010-2011. | | | | | | | | | | |
| --- | --- | --- | --- | --- | --- | --- | --- | --- | --- | --- |
|  | Males (15-29) | | | | | Females (15-29) | | | | |
|  | S. ctrl | UCT | UCT vs. s. ctrl (reference) | | | S. ctrl | UCT | UCT vs. s. ctrl (reference) | | |
| Outcome: | n/N (%) | n/N (%) | ATE | (95% CI) | p-value | n/N (%) | n/N (%) | ATE | (95% CI) | p-value |
| **Primary outcomes** | | | | | | | | | | |
| Had sexual debut ^[a]^ | 18/196 (9.14) | 12/188 (6.38) | -2.30 | (-7.27, 2.66) | 0.363 | 29/157 (18.5) | 31/156 (19.9) | -0.24 | (-5.08, 4.59) | 0.921 |
| Had sex in past 30 days | 42/60 (72.2) | 36/60 (61.7) | -10.5 | (-20.9, -0.03) | 0.049 | 100/137 (71.5) | 98/137 (69.9) | -1.66 | (-13.8, 10.5) | 0.789 |
| Condom use (last sex) | 19/60 (30.4) | 21/60 (32.8) | 2.33 | (-8.50, 13.2) | 0.673 | 11/137 (7.86) | 22/137 (16.0) | 8.10 | (1.20, 15.0) | 0.021 |
| Multiple partners (past 12 months) | 8/60 (12.9) | 14/60 (24.5) | 11.6 | (-9.65, 32.8) | 0.285 | 0/124 (0.00) | 0/124 (0.00) | NA ^[b]^ |  |  |
| Transactional sex ^[c]^ | 2/60 (2.69) | 2/60 (2.66) | -0.03 | (-6.74, 6.68) | 0.993 | 0/124 (0.00) | 0/124 (0.00) | NA ^[b]^ |  |  |
| Has a partner 5+ years older | 14/59 (25.1) | 18/59 (31.2) | 6.12 | (-9.42, 21.7) | 0.440 | 64/124 (51.7) | 79/124 (63.7) | 12.0 | (1.07, 22.9) | 0.031 |
| **Secondary outcomes** | | | | | | | | | | |
| Psychological distress ^[d]^ | 36/261 (13.1) | 30/261 (10.8) | -2.36 | (-7.65, 2.92) | 0.381 | 46/267 (16.8) | 64/267 (23.6) | 6.76 | (-2.01, 15.5) | 0.131 |
| Self-efficacy ^[e]^ | 211/261 (81.1) | 209/261 (80.4) | -0.77 | (-7.87, 6.34) | 0.833 | 233/267 (87.1) | 218/267 (81.7) | -5.44 | (-9.62, -1.25) | 0.011 |
| Risk perception ^[f]^ | 25/261 (9.74) | 19/261 (7.22) | -2.52 | (-8.85, 3.82) | 0.436 | 65/256 (25.9) | 58/256 (22) | -3.87 | (-8.29, 0.56) | 0.087 |
| Had HIV test in past 12 months | 31/261 (11.1) | 35/261 (12.5) | 1.41 | (-2.56, 5.37) | 0.487 | 93/267 (35.1) | 110/267 (40.5) | 5.41 | (-4.67, 15.5) | 0.293 |
| Is currently enrolled in school ^[a]^ | 123/196 (64.4) | 135/188 (71.9) | 7.46 | (-0.84, 15.8) | 0.078 | 91/157 (57.3) | 98/156 (63.3) | 6.00 | (0.16, 11.8) | 0.044 |
| Alcohol use ^[g]^ | 32/261 (12.5) | 33/261 (12.6) | 0.08 | (-5.61, 5.77) | 0.979 | 2/267 (0.86) | 1/267 (0.38) | -0.48 | (-1.86, 0.90) | 0.495 |
| Smokes cigarettes | 16/260 (6.18) | 20/260 (7.69) | 1.51 | (-2.70, 5.72) | 0.481 | 0/267 (0.00) | 0/267 (0.00) | NA ^[b]^ |  |  |
| Takes recreational drugs | 24/260 (8.54) | 22/260 (6.77) | -1.77 | (-8.15, 4.60) | 0.586 | 0/267 (0.00) | 0/267 (0.00) | NA ^[b]^ |  |  |
| S. ctrl: Synthetic control; UCT: Unconditional cash transfer; ATE: Average treatment effect; CI: Confidence interval  Sample: Young people (15-29 years) who had sex before for primary outcomes and all young people for secondary outcomes. The synthetic comparison group was determined through propensity score matching of individuals from the Manicaland Cohort to treatment-group individuals from the Manicaland Trial.  Numbers are sample sizes of individuals reporting the outcome among everyone with data on the outcome (n/N) together with percentages (%) in the synthetic control and treatment (UCT) groups of the Manicaland Trial and the estimated ATEs with 95% CIs and p-values. ATEs for each for each outcome were estimated from separate mixed-effects logistic regression model after propensity score matching, controlling for age and wealth index quarters (not shown), with study site and treatment cluster random effects. Propensity score matching for the control group was implemented with replacement. Probability weights were applied, so individuals may be counted several times, and numbers were rounded.  ^[a]^ Analyses were restricted to those aged 15-20 years as it was not applicable to older individuals.  ^[b]^ No regression model was estimated due to sample size limitations.  ^[c]^ For males: Given any financial or material reward for sex in the past 12 months. For females: Received any financial or material reward for sex in the past 12 months.  ^[d]^ Reporting at least 7 symptoms of psychological distress of a 25-item scale.  ^[e]^ Reporting that there are things that can be done to prevent HIV infection.  ^[f]^ Perceiving a risk for HIV infection in the future.  ^[g]^ Having been to a beer hall (bar) in the past month or drinking more than 3 drinks when drinking alcohol. | | | | | | | | | | |

## 3. Additional results by type of sex of head of household

In this section, additional results for analyses by sex of the head of household are provided. Table S3.9 provides a detailed distribution of individuals by sex of head of household and whether they are heads of households themselves. The main article presents results without sample sizes and p-values. Table S3.10 and Table S3.11 provide full results for effects of CTs among young people by sex of head of household. Table S3.12 and Table S3.13 provide full results for older people.

| Table S3.9 Distribution of individuals in male- and female-headed households receiving cash transfers (treatment) and relationships to head of household, Manicaland Cash Transfer Trial, Manicaland, Zimbabwe, 2010-11. | | | | | |
| --- | --- | --- | --- | --- | --- |
|  |  | Younger people (15-29) | | Older people (30-54) | |
|  | Total N (%) | Males N (%) | Females N (%) | Males N (%) | Females N (%) |
| Type of household |  |  |  |  |  |
| Male-headed | 1050 (51.2) | 250 (46.7) | 301 (52.3) | 214 (88.1) | 285 (40.9) |
| Female-headed | 1001 (48.8) | 285 (53.3) | 275 (47.7) | 29 (11.9) | 412 (59.1) |
|  |  |  |  |  |  |
| Relationship to head of household |  |  |  |  |  |
| Head of household | 631 (30.7) | 34 (6.36) | 29 (5.02) | 203 (83.5) | 365 (52.1) |
| Spouse | 405 (19.7) | 2 (0.37) | 125 (21.6) | 14 (5.76) | 264 (37.7) |
| Child/grandchild | 877 (42.6) | 439 (82.1) | 362 (62.6) | 22 (9.05) | 54 (7.70) |
| Other relative | 144 (7.00) | 60 (11.2) | 62 (10.7) | 4 (1.65) | 18 (2.57) |
| Values are sample sizes (N) and percentages (%). | | | | | |

| Table S3.10 Effects of cash transfers on young males (15-29 years) in male- and female-headed households, Manicaland Cash Transfer Trial, Manicaland, Zimbabwe, 2010-2011. | | | | | | | | | | |
| --- | --- | --- | --- | --- | --- | --- | --- | --- | --- | --- |
|  | Male-headed households | | | | | Female-headed households | | | | |
|  | Ctrl | CT | CT vs. ctrl (reference) | | | Ctrl | CT | CT vs. ctrl (reference) | | |
| Outcome: | n/N (%) | n/N (%) | ATE | (95% CI) | p-value | n/N (%) | n/N (%) | ATE | (95% CI) | p-value |
| **Primary outcomes** | | | | | | | | | | |
| Had sexual debut ^[a]^ | 2/60 (3.33) | 15/174 (8.62) | 5.18 | (-0.59, 10.9) | 0.079 | 5/96 (5.21) | 13/217 (5.99) | -1.58 | (-8.55, 5.38) | 0.656 |
| Had sex in past 30 days | 29/34 (85.3) | 40/67 (59.7) | -20.0 | (-39.1, -0.9) | 0.040 | 12/23 (52.2) | 29/55 (52.7) | 1.29 | (-21.7, 24.3) | 0.912 |
| Condom use (last sex) | 9/34 (26.5) | 27/67 (40.3) | 10.9 | (-8.41, 30.1) | 0.269 | 12/23 (52.2) | 24/55 (43.6) | -12.9 | (-35.5, 9.76) | 0.265 |
| Multiple partners (past 12 months) | 7/34 (20.6) | 19/67 (28.4) | 3.30 | (-16.5, 23.1) | 0.744 | 3/23 (13.0) | 12/55 (21.8) | 6.92 | (-11.9, 25.7) | 0.470 |
| Transactional sex ^[n]^ | 2/34 (5.88) | 4/66 (6.06) | 0.31 | (-7.94, 8.57) | 0.941 | 1/23 (4.35) | 0/55 (0.00) | NA ^[c]^ |  |  |
| **Secondary outcomes** | | | | | | | | | | |
| Psychological distress ^[d]^ | 12/103 (11.7) | 32/250 (12.8) | 1.27 | (-6.28, 8.82) | 0.741 | 17/128 (13.3) | 26/285 (9.12) | -4.55 | (-12.8, 3.72) | 0.281 |
| Self-efficacy ^[e]^ | 87/103 (84.5) | 198/250 (79.2) | -6.45 | (-17.7, 4.79) | 0.261 | 100/128 (78.1) | 238/285 (83.5) | 5.40 | (-3.00, 13.8) | 0.205 |
| Risk perception ^[f]^ | 12/103 (11.7) | 21/250 (8.40) | -1.53 | (-8.50, 5.43) | 0.666 | 16/126 (12.7) | 27/284 (9.51) | -1.86 | (-9.94, 6.22) | 0.652 |
| Had HIV test in past 12 months | 17/103 (16.5) | 30/250 (12.0) | 0.14 | (-7.97, 8.26) | 0.972 | 23/128 (18.0) | 36/284 (12.7) | -5.61 | (-13.4, 2.20) | 0.159 |
| Is currently enrolled in school ^[a]^ | 34/60 (56.7) | 124/174 (71.3) | 17.9 | (6.65, 29.1) | 0.002 | 67/96 (69.8) | 157/217 (72.4) | 8.28 | (-2.68, 19.2) | 0.139 |
| Alcohol use ^[g]^ | 21/103 (20.4) | 35/250 (14.0) | 0.60 | (-6.70, 7.91) | 0.871 | 13/128 (10.2) | 29/285 (10.2) | -1.17 | (-6.89, 4.55) | 0.689 |
| Smokes cigarettes | 11/103 (10.7) | 18/250 (7.20) | 0.07 | (-5.69, 5.83) | 0.982 | 9/128 (7.03) | 18/284 (6.34) | -2.48 | (-7.70, 2.75) | 0.353 |
| Takes recreational drugs | 16/103 (15.5) | 22/250 (8.8) | -3.50 | (-12.1, 5.05) | 0.422 | 10/128 (7.81) | 25/283 (8.83) | -0.18 | (-6.23, 5.86) | 0.953 |
| Ctrl: Control; CT: Cash transfer; ATE: Average treatment effect; CI: Confidence interval  Sample: Young males (15-29 years) who had sex before for primary outcomes and all young people for secondary outcomes, restricted to male- and female-headed households, respectively.  Numbers are sample sizes of individuals reporting the outcome among everyone with data on the outcome (n/N) together with percentages (%) in the synthetic control and treatment (CT) groups of the Manicaland Trial and the estimated ATEs with 95% CIs and p-values, separately for males in male- or female-headed households. ATEs for each outcome were estimated from separate mixed-effects logistic regression model, controlling for age and wealth index quarters (not shown), with study site and treatment cluster random effects.  ^[a]^ Analyses were restricted to those aged 15-20 years as it was not applicable to older individuals.  ^[b]^ For males: Given any financial or material reward for sex in the past 12 months. For females: Received any financial or material reward for sex in the past 12 months.  ^[c]^ No regression model was estimated due to sample size limitations.  ^[d]^ Reporting at least 7 symptoms of psychological distress of a 25-item scale.  ^[e]^ Reporting that there are things that can be done to prevent HIV infection.  ^[f]^ Perceiving a risk for HIV infection in the future.  ^[g]^ Having been to a beer hall (bar) in the past month or drinking more than 3 drinks when drinking alcohol. | | | | | | | | | | |

| Table S3.11 Effects of cash transfers on young females (15-29 years) in male- and female-headed households, Manicaland Cash Transfer Trial, Manicaland, Zimbabwe, 2010-2011. | | | | | | | | | | |
| --- | --- | --- | --- | --- | --- | --- | --- | --- | --- | --- |
|  | Male-headed households | | | | | Female-headed households | | | | |
|  | Ctrl | CT | CT vs. ctrl (reference) | | | Ctrl | CT | CT vs. ctrl (reference) | | |
| Outcome: | n/N (%) | n/N (%) | ATE | (95% CI) | p-value | n/N (%) | n/N (%) | ATE | (95% CI) | p-value |
| **Primary outcomes** | | | | | | | | | | |
| Had sexual debut ^[a]^ | 11/57 (19.3) | 32/152 (21.1) | 4.56 | (-5.58, 14.7) | 0.378 | 9/69 (13) | 33/196 (16.8) | -0.09 | (-9.45, 9.27) | 0.985 |
| Had sex in past 30 days | 61/71 (85.9) | 143/180 (79.4) | -7.57 | (-18.1, 2.92) | 0.157 | 21/39 (53.9) | 53/101 (52.5) | 2.51 | (-16.6, 21.6) | 0.797 |
| Condom use (last sex) | 9/71 (12.7) | 25/180 (13.9) | 1.74 | (-7.40, 10.9) | 0.709 | 10/39 (25.6) | 21/101 (20.8) | -2.52 | (-20.6, 15.5) | 0.784 |
| Has a partner 5+ years older | 42/70 (60.0) | 110/172 (64.0) | 4.48 | (-9.02, 18.0) | 0.515 | 23/39 (59) | 53/91 (58.2) | 0.22 | (-19.0, 19.4) | 0.982 |
| **Secondary outcomes** | | | | | | | | | | |
| Psychological distress ^[b]^ | 33/120 (27.5) | 70/301 (23.3) | -3.87 | (-12.9, 5.2) | 0.403 | 19/105 (18.1) | 46/275 (16.7) | 0.34 | (-8.18, 8.86) | 0.938 |
| Self-efficacy ^[c]^ | 95/120 (79.2) | 246/301 (81.7) | 1.24 | (-8.31, 10.8) | 0.799 | 81/105 (77.1) | 234/275 (85.1) | 7.77 | (-3.96, 19.5) | 0.194 |
| Risk perception ^[d]^ | 36/115 (31.3) | 74/289 (25.6) | -5.74 | (-18.1, 6.56) | 0.360 | 11/105 (10.5) | 56/267 (21.0) | 11.6 | (4.13, 19.1) | 0.002 |
| Had HIV test in past 12 months | 58/120 (48.3) | 125/300 (41.7) | -5.79 | (-17.0, 5.46) | 0.313 | 40/105 (38.1) | 95/275 (34.6) | -0.10 | (-9.78, 9.58) | 0.984 |
| Is currently enrolled in school ^[a]^ | 36/57 (63.2) | 89/152 (58.6) | -8.94 | (-20.1, 2.19) | 0.115 | 46/69 (66.7) | 137/196 (69.9) | 5.15 | (-6.48, 16.8) | 0.386 |
| Ctrl: Control; CT: Cash transfer; ATE: Average treatment effect; CI: Confidence interval  Sample: Young females (15-29 years) who had sex before for primary outcomes and all young people for secondary outcomes, restricted to male- and female-headed households, respectively.  Numbers are sample sizes of individuals reporting the outcome among everyone with data on the outcome (n/N) together with percentages (%) in the synthetic control and treatment (CT) groups of the Manicaland Trial and the estimated ATEs with 95% CIs and p-values, separately for females in male- or female-headed households. ATEs for each outcome were estimated from separate mixed-effects logistic regression model, controlling for age and wealth index quarters (not shown), with study site and treatment cluster random effects. No results for multiple partners, transactional sex, alcohol use, cigarette smoking, and drug consumption are shown because of very small sample sizes.  ^[a]^ Analyses were restricted to those aged 15-20 years as it was not applicable to older individuals.  ^[b]^ Reporting at least 7 symptoms of psychological distress of a 25-item scale.  ^[c]^ Reporting that there are things that can be done to prevent HIV infection.  ^[d]^ Perceiving a risk for HIV infection in the future. | | | | | | | | | | |

| Table S3.12 Effects of cash transfers on older males (30-54 years) in male- and female-headed households, Manicaland Cash Transfer Trial, Manicaland, Zimbabwe, 2010-2011. | | | | | | | | | | |
| --- | --- | --- | --- | --- | --- | --- | --- | --- | --- | --- |
|  | Male-headed households | | | | | Female-headed households | | | | |
|  | Ctrl | CT | CT vs. ctrl (reference) | | | Ctrl | CT | CT vs. ctrl (reference) | | |
| Outcome: | n/N (%) | n/N (%) | ATE | (95% CI) | p-value | n/N (%) | n/N (%) | ATE | (95% CI) | p-value |
| **Primary outcomes** | | | | | | | | | | |
| Had sex in past 30 days | 73/79 (92.4) | 184/212 (86.8) | -6.29 | (-13.9, 1.33) | 0.105 | 11/13 (84.6) | 23/27 (85.2) | 8.62 | (-16.6, 33.8) | 0.502 |
| Condom use (last sex) | 17/79 (21.5) | 41/212 (19.3) | -0.76 | (-11.7, 10.2) | 0.891 | 5/13 (38.5) | 7/27 (25.9) | -15.7 | (-46.7, 15.4) | 0.324 |
| Multiple partners (past 12 months) | 13/79 (16.5) | 20/213 (9.39) | -6.29 | (-15.6, 3.04) | 0.186 | 0/13 (0.00) | 4/27 (14.8) | NA ^[a]^ |  |  |
| Transactional sex ^[b]^ | 3/78 (3.85) | 6/213 (2.82) | 0.04 | (-3.47, 3.55) | 0.983 | 0/13 (0.00) | 0/27 (0.00) | NA ^[a]^ |  |  |
| **Secondary outcomes** | | | | | | | | | | |
| Psychological distress ^[c]^ | 14/81 (17.3) | 36/214 (16.8) | -4.58 | (-17.3, 8.15) | 0.480 | 2/13 (15.4) | 5/29 (17.2) | 4.08 | (-18.1, 26.3) | 0.719 |
| Self-efficacy ^[d]^ | 63/81 (77.8) | 146/214 (68.2) | -10.6 | (-24.9, 3.75) | 0.148 | 10/13 (76.9) | 21/29 (72.4) | -7.31 | (-34.2, 19.6) | 0.594 |
| Risk perception ^[e]^ | 12/69 (17.4) | 17/191 (8.90) | -8.33 | (-19.9, 3.24) | 0.158 | 0/11 (0.00) | 2/27 (7.41) | NA ^[a]^ |  |  |
| Had HIV test in past 12 months | 27/81 (33.3) | 58/213 (27.2) | -5.31 | (-19.6, 8.95) | 0.466 | 3/13 (23.1) | 10/29 (34.5) | 11.2 | (-18.1, 40.4) | 0.455 |
| Alcohol use ^[f]^ | 31/81 (38.3) | 90/214 (42.1) | 5.27 | (-10.2, 20.8) | 0.506 | 7/13 (53.9) | 14/29 (48.3) | -6.27 | (-39.4, 26.8) | 0.710 |
| Smokes cigarettes | 27/81 (33.3) | 68/214 (31.8) | -2.52 | (-15.7, 10.7) | 0.708 | 5/13 (38.5) | 12/29 (41.4) | 4.08 | (-28.0, 36.2) | 0.803 |
| Takes recreational drugs | 29/81 (35.8) | 54/214 (25.2) | -9.64 | (-23.8, 4.54) | 0.183 | 6/13 (46.2) | 9/28 (32.1) | -10.3 | (-43.7, 23.1) | 0.547 |
| Ctrl: Control; CT: Cash transfer; ATE: Average treatment effect; CI: Confidence interval  Sample: Older males (30-54 years) who had sex before for primary outcomes and all older people for secondary outcomes, restricted to male- and female-headed households, respectively.  Numbers are sample sizes of individuals reporting the outcome among everyone with data on the outcome (n/N) together with percentages (%) in the control and treatment (CT) groups of the Manicaland Trial and the estimated ATEs with 95% CIs and p-values, separately for males in male- or female-headed households. ATEs for each outcome were estimated from separate mixed-effects logistic regression model, controlling for age and wealth index quarters (not shown), with study site and treatment cluster random effects.  ^[a]^ No regression model was estimated due to sample size limitations.  ^[b]^ For males: Given any financial or material reward for sex in the past 12 months. For females: Received any financial or material reward for sex in the past 12 months.  ^[c]^ Reporting at least 7 symptoms of psychological distress of a 25-item scale.  ^[d]^ Reporting that there are things that can be done to prevent HIV infection.  ^[e]^ Perceiving a risk for HIV infection in the future.  ^[f]^ Having been to a beer hall (bar) in the past month or drinking more than 3 drinks when drinking alcohol. | | | | | | | | | | |

| Table S3.13 Effects of cash transfers on older females (30-54 years) in male- and female-headed households, Manicaland Cash Transfer Trial, Manicaland, Zimbabwe, 2010-2011. | | | | | | | | | | |
| --- | --- | --- | --- | --- | --- | --- | --- | --- | --- | --- |
|  | Male-headed households | | | | | Female-headed households | | | | |
|  | Ctrl | CT | CT vs. ctrl (reference) | | | Ctrl | CT | CT vs. ctrl (reference) | | |
| Outcome: | n/N (%) | n/N (%) | ATE | (95% CI) | p-value | n/N (%) | n/N (%) | ATE | (95% CI) | p-value |
| **Primary outcomes** | | | | | | | | | | |
| Had sex in past 30 days | 96/113 (85.0) | 229/283 (80.9) | -4.28 | (-12.2, 3.60) | 0.287 | 56/187 (30.0) | 102/409 (24.9) | -3.94 | (-11.6, 3.71) | 0.313 |
| Condom use (last sex) | 16/113 (14.2) | 55/283 (19.4) | 6.33 | (-1.53, 14.2) | 0.115 | 51/187 (27.3) | 105/408 (25.7) | -1.14 | (-8.67, 6.38) | 0.766 |
| **Secondary outcomes** | | | | | | | | | | |
| Psychological distress ^[a]^ | 46/114 (40.4) | 103/285 (36.1) | -6.12 | (-18.1, 5.82) | 0.315 | 72/187 (38.5) | 150/412 (36.4) | -2.47 | (-11.9, 6.96) | 0.607 |
| Self-efficacy ^[b]^ | 92/114 (80.7) | 223/285 (78.3) | -2.72 | (-11.4, 5.96) | 0.539 | 128/187 (68.5) | 263/412 (63.8) | -4.33 | (-13.2, 4.58) | 0.341 |
| Risk perception ^[c]^ | 46/106 (43.4) | 116/253 (45.9) | 2.67 | (-9.75, 15.1) | 0.674 | 54/148 (36.5) | 104/322 (32.3) | -3.85 | (-16.3, 8.64) | 0.546 |
| Had HIV test in past 12 months | 60/114 (52.6) | 150/285 (52.6) | 0.60 | (-10.3, 11.5) | 0.915 | 85/187 (45.5) | 187/412 (45.4) | 0.62 | (-9.83, 11.2) | 0.907 |
| Ctrl: Control; CT: Cash transfer; ATE: Average treatment effect; CI: Confidence interval  Sample: Older females (30-54 years) who had sex before for primary outcomes and all older people for secondary outcomes, restricted to male- and female-headed households, respectively.  Numbers are sample sizes of individuals reporting the outcome among everyone with data on the outcome (n/N) together with percentages (%) in the control and treatment (CT) groups of the Manicaland Trial and the estimated ATEs with 95% CIs and p-values, separately for females in male- or female-headed households. ATEs for each outcome were estimated from separate mixed-effects logistic regression model, controlling for age and wealth index quarters (not shown), with study site and treatment cluster random effects. No results for multiple partners, transactional sex, alcohol use, cigarette smoking, and drug consumption are shown because of very small sample sizes.  ^[a]^ Reporting at least 7 symptoms of psychological distress of a 25-item scale.  ^[b]^ Reporting that there are things that can be done to prevent HIV infection.  ^[c]^ Perceiving a risk for HIV infection in the future. | | | | | | | | | | |
